# Biotin pathway in novel *Fodinibius salsisoli* sp. nov., isolated from hypersaline soils and reclassification of the genus *Aliifodinibius* as *Fodinibius*

**DOI:** 10.3389/fmicb.2022.1101464

**Published:** 2023-01-26

**Authors:** Cristina Galisteo, Rafael R. de la Haba, Cristina Sánchez-Porro, Antonio Ventosa

**Affiliations:** Department of Microbiology and Parasitology, Faculty of Pharmacy, University of Sevilla, Sevilla, Spain

**Keywords:** *Balneolaceae*, hypersaline soils, phylogenomics, biotin biosynthesis, genomic analysis, moderate halophile, taxonomic reclassification

## Abstract

Hypersaline soils are extreme environments that have received little attention until the last few years. Their halophilic prokaryotic population seems to be more diverse than those of well-known aquatic systems. Among those inhabitants, representatives of the family *Balneolaceae* (phylum *Balneolota*) have been described to be abundant, but very few members have been isolated and characterized to date. This family comprises the genera *Aliifodinibius* and *Fodinibius* along with four others. A novel strain, designated 1BSP15-2V2^T^, has been isolated from hypersaline soils located in the Odiel Saltmarshes Natural Area (Southwest Spain), which appears to represent a new species related to the genus *Aliifodinibius*. However, comparative genomic analyses of members of the family *Balneolaceae* have revealed that the genera *Aliifodinibius* and *Fodinibius* belong to a single genus, hence we propose the reclassification of the species of the genus *Aliifodinibius* into the genus *Fodinibius,* which was first described. The novel strain is thus described as *Fodinibius salsisoli* sp. nov., with 1BSP15-2V2^T^ (=CCM 9117^T^ = CECT 30246^T^) as the designated type strain. This species and other closely related ones show abundant genomic recruitment within 80–90% identity range when searched against several hypersaline soil metagenomic databases investigated. This might suggest that there are still uncultured, yet abundant closely related representatives to this family present in these environments. In-depth *in-silico* analysis of the metabolism of *Fodinibius* showed that the biotin biosynthesis pathway was present in the genomes of strain 1BSP15-2V2^T^ and other species of the family *Balneolaceae*, which could entail major implications in their community role providing this vitamin to other organisms that depend on an exogenous source of this nutrient.

## 1. Introduction

The family *Balneolaceae* (Xia et al., 2016) belongs to the phylum *Balneolota*, class *Balneolia* (Munoz et al., 2016), and includes six genera: *Aliifodinibius*, *Balneola*, *Fodinibius*, *Gracilimonas*, *Halalkalibaculum*, and *Rhodohalobacter* (Parte et al., 2020), which are found in marine and hypersaline environments, such as deep-sea sediments, salt-mines, salterns, and saline soils (Urios et al., 2006; Choi et al., 2009; Wang et al., 2012, 2013; Cho et al., 2013; Xia et al., 2017; Wu et al., 2022a,b). The genus *Fodinibius* was proposed in 2012 based on a single species, *Fodinibius salinus* (Wang et al., 2012) and the genus *Aliifodinibius* was firstly described only one year later (Wang et al., 2013), including at that time two species, *Aliifodinibius roseus* (type species) and *Aliifodinibius sediminis*. These taxa shared low 16S rRNA gene sequence identities (<92.4%) with *F. salinus,* the most closely related species. However, no further genomic comparison was performed at that time. The aforementioned identity values, along with their differential fatty acids and polar lipids profiles, as well as some phenotypic features, were considered enough evidence to propose that *A. roseus* and *A. sediminis* represented a novel genus, different from *Fodinibius*. At the time of writing, five species names have been validly published within the genus *Aliifodinibius*: *Aliifodinibius roseus* and *Aliifodinibius sediminis* (Wang et al., 2013), *Aliifodinibius halophilus* (Xia et al., 2016), *Aliifodinibius salicampi* (Cho et al., 2017, 2018), and *Aliifodinibius saliphilus* (Cho and Whang, 2020). Besides, the species “*Aliifodinibius salipaludis*” (Zhao et al., 2020) has been described but its name has not been validly published so far. All these species were isolated from hypersaline environments, such as salt mines, salterns, and saline soils. They are Gram-stain-negative, rod-shaped, non-motile, and moderately halophilic bacteria, with a NaCl requirement for growing between 4 and 25% (w/v) and an optimum between 8 and 15%, except for “*A. salipaludis*,” that can grow optimally at 25% (w/v) NaCl (Zhao et al., 2020). The pH values supporting their growth range between pH 6.0 and 11.0, with an optimum at pH 7.0–8.0. They are able to produce catalase and oxidase but unable to hydrolyze starch and DNA (Wang et al., 2013; Xia et al., 2016; Cho et al., 2017; Cho and Whang, 2020; Zhao et al., 2020).

The Odiel Saltmarshes Natural Area is located in the estuary of the Odiel and Tinto rivers (Southwest Spain), and its hypersaline soils have been previously explored by Vera-Gargallo and Ventosa (2018). They obtained two metagenomes database of these area named SMO1 and SMO2. Their shotgun metagenomic analysis taxonomically annotated almost a 20% of the prokaryotic diversity as representatives of the phylum *Balneolota*. Genus-level taxonomic affiliation of 16S rRNA genes detected in the reads above 95% identity assigned 9.0 and 3.4% of the abundance to the genera *Fodinibius* and *Gracilimonas*, respectively, for the SMO1 metagenomic database, and 3.0 and 1.6% for the SMO2 metagenomic database. Other studies have also revealed the presence of this taxonomic group on different metagenomic datasets. The abundance of the uncultured members of the class *Balneolia* was up to 8% in the hypersaline soda lake sediment studied by Vavourakis et al. (2018), being one of the three predominant bacterial groups along with the phylum *Bacillota* and the class *Gammaproteobacteria*. Both studies recovered MAGs related to uncultured members of the family *Balneolaceae* (Vavourakis et al., 2018; Vera-Gargallo and Ventosa, 2018). Kimbrel et al. (2018) obtained a medium-quality MAG related to *Gracilimonas tropica*, with a 0.26% of abundance, from sediments with salinity of 7.5%. The thalassohaline brines located in Maras (Peru) showed a considerable abundance of up to 6% of *Balneolota*, mainly associated (5% out of the total) to the species *A. roseus* (Castelán-Sánchez et al., 2019). These previous studies indicate that the members of the family *Balneolaceae* are fairly abundant in hypersaline ecosystems, although the number of cultivated species is still scarce.

In the present study, we describe the isolation of a new strain, following a culturomic strategy, and we have carried out its exhaustive description as a novel species within the family *Balneolaceae*. Besides, we completed the phylogenomic analyses of the whole family, which revealed the need for merging two of its genera. Furthermore, we performed genome-based functional analyses of these bacteria, including the vitamin B_7_ pathway, to elucidate their potential ecological role and adaptative mechanisms to survive under harsh conditions of salinity and heavy metals, along with its distribution in hypersaline habitats.

## 2. Materials and methods

### 2.1. Strain isolation and physico-chemical features of the sampling site

The samples were collected from hypersaline soils located at the saltmarshes of the Odiel Natural Area in Huelva (Southwest Spain) as previously described (Vera-Gargallo and Ventosa, 2018; Vera-Gargallo et al., 2019). The pH and electrical conductivity of the saline soil was measured with the pHmeter CRISON BASIC 20 and the conductometer CRISON 35+, respectively, after a 1:5 dilution. Arsenic and cadmium concentrations were measured by Inductively Coupled Plasma Mass Spectrometry (ICP-MS); copper and zinc concentrations, by Inductively Coupled Plasma Optical Emission Spectroscopy (ICP-OES); and lead concentration, by atomic absorption spectroscopy. All those spectrometric assays were carried out by Innoagral Laboratories (Mairena del Aljarafe, Sevilla, Spain).

The strain 1BSP15-2V2^T^ was isolated using the standard dilution-plating technique and grew on a low-nutrient medium enriched with pyruvate (SMM medium) at 28°C after 3 months of incubation. The colonies were subcultured until a pure culture was obtained. The composition of the low nutrients SMM medium was the following (g L^−1^): casein digest, 5.0; sodium pyruvate, 1.1; supplemented with up to 15% (w/v) salt concentration from a seawater (SW) stock ([g L^−1^] NaCl, 234.0; MgCl_2_·6H_2_O, 39.0; MgSO_4_·7H_2_O, 61.0; CaCl_2_, 1.0; KCl, 6.0; NaHCO_3_, 0.2; NaBr, 0.7); the pH was adjusted to 7.5; solidified with 2.0% (w/v) agar when needed. This medium was first used by León et al. (2016) to isolate the species of the genus *Spiribacter*, which is an abundant halophilic bacterium of the salterns community at intermediate salinities. The culture was preserved at −80°C in the same liquid medium with 40% (v/v) glycerol. Additionally, Marine Agar (MA, Difco 2216) medium, supplemented with 10% (w/v) NaCl, was used to determine the fatty acid profile of this strain for better comparative purposes with other species of the genus under study. In addition, reference type strains of the species of the genus *Aliifodinibius* were used for comparative phenotypic analyses. The following strains were obtained from the corresponding culture collections: *A. halophilus* KCTC 42497^T^, *A. roseus* DSM 21986^T^, *A. salicampi* KACC 19060^T^, “*A. salipaludis*” KCTC 52855^T^, *A. saliphilus* KACC 19126^T^, and *A. sediminis* DSM 21194^T^. These strains were grown and preserved in R2A medium supplemented with 7.5% (w/v) SW. Additionally, DNA of strain *A. salicampi* KACC 19060^T^ was extracted and sequenced as detailed below as it was the only species of the genus *Aliifodinibius* whose genome was not publicly available.

### 2.2. DNA extraction and PCR amplification

Genomic DNA of strain 1BSP15-2V2^T^ was extracted using the Marmur (1961) method modified for small volumes. The almost complete 16S rRNA gene sequence was amplified by PCR with universal primers 27F (5′-AGA GTT TGA TCM TGG CTC AG-3′) and 1492R (5′-GGT TAC CTT GTT ACG ACT T3′) (Lane, 1991). Both genomic DNA and PCR products were purified using MEGAquick-spin™ plus (iNtRON Biotechnology), following the manufacturer’s recommendations. The 16S rRNA gene was sequenced using Sanger technology by StabVida (Caparica, Portugal). The same protocol was followed for the DNA extraction of strain *A. salicampi* KACC 19060^T^.

### 2.3. Phylogenetic analysis

Identification of phylogenetic neighbors of strain 1BSP15-2V2^T^ according to the 16S rRNA gene sequence identity was carried out searching the EzBioCloud database^1^ (Yoon et al., 2017). For further phylogenetic analysis, the type strains of the identified closely related taxa of the family *Balneolaceae* were used. The 16S rRNA gene sequences of the strains included in the analysis were obtained from SILVA and GenBank databases. Alignment of the primary and secondary structures of the selected 16S rRNA sequences was performed using the integrated fast aligner implemented in the ARB package (Ludwig et al., 2004). For phylogenetic tree reconstruction, maximum-likelihood (Felsenstein, 1981), maximum-parsimony (Felsenstein, 1983), and neighbor-joining (Saitou and Nei, 1987) algorithms integrated in the ARB software (Ludwig et al., 2004) were used, and the Jukes-Cantor model (Jukes and Cantor, 1969) was selected to correct the distance matrix. The robustness of the branch topology was checked by bootstrap analysis (1,000 pseudoreplicates) (Felsenstein, 1985). The script “gitana”^2^ was employed for formatting and imaging of the tree visualization.

### 2.4. Comparative genomic analyses

Whole shotgun sequencing of the genome of strain 1BSP15-2V2^T^ was performed by Novogene Europe (Cambridge, United Kingdom) on the Illumina NovaSeq PE150 platform. Paired end reads were assembled with SPAdes v3.13.0 (Prjibelski et al., 2020). Quality control of the assembly was performed with QUAST v2.3 (Gurevich et al., 2013), which also calculated some genomic statistics, such as the DNA G + C content. The length-filtered scaffolds were screened by using Prodigal v2.60 (Hyatt et al., 2010), to extract the nucleotide coding sequences as well as their protein translation, and Prokka v1.12 (Seemann, 2014) for the standard GenBank annotation. CheckM v1.0.5 (Parks et al., 2015) allow us to verify genome completeness and contamination. The same pipeline was followed for the genome sequencing and analysis of strain *A. salicampi* KACC 19060^T^. Additionally, the available draft genomes of the type strains of species of the genera *Aliifodinibius*, *Balneola*, *Fodinibius*, *Gracilimonas, Halalkalibaculum,* and *Rhodohalobacter* were used for comparative genomic analyses (Supplementary Table S1).

Isoelectric point calculation of predicted proteins was carried out using the “iep” program implemented in the EMBOSS package v6.5.7.0 (Rice et al., 2000), and plotted with the R package “ggplot2” v3.3.3 (Wickham, 2009). Meanwhile, the R package “fmsb” v0.7.3 (Nakazawa, 2022) was used for displaying the spider (or radar) graph of amino acid frequency. In both cases, colors were chosen from the wide selection of the R package “paletteer” v.1.4.0 (Hvitfeldt, 2021). Functional annotation was performed with the online tool BlastKOALA (Kanehisa et al., 2016). The KEGG Orthology numbers (KO), corresponding to KEGG pathways and KEGG modules, were assigned to the identified coding sequences, and then plotted in a heatmap to identify the most abundant functions. KO definitions were manually inspected and compared among the species of the family *Balneolaceae*.

Following the proposed minimal standards for prokaryotic taxonomy (Chun et al., 2018), we used different Overall Genome Relatedness Indexes (OGRIs) to determine the status of strain 1BSP15-2V2^T^ within the family *Balneolaceae*. Average Amino acid Identity (AAI) and Average Nucleotide Identity for orthologous sequences (orthoANI) were calculated using the Enveomics toolbox (Rodriguez-R and Konstantinidis, 2016) and OAU software v1.2 (Lee et al., 2016), respectively. For digital DNA–DNA hybridization (dDDH), the Genome-to-Genome Distance Calculator bioinformatic tool (GGDC v3.0) available from the Leibniz Institute DSMZ (Meier-Kolthoff et al., 2021) was used. For a more reliable determination of the relationship among the members of the *Balneolaceae*, a phylogenomic tree based on the core-proteome sequences of those strains was built. The orthologous protein clusters were identified after BLASTp v2.2.28+ search and extracted from the proteome by using the Markov Cluster Algorithm, as implemented in the Enveomics toolbox (Rodriguez-R and Konstantinidis, 2016). Muscle v3.8.31 (Edgar, 2004) was employed to align the orthologous sequences. The phylogeny was calculated based on 1,049 concatenated protein sequences with the approximately maximum-likelihood algorithm implemented in FastTreeMP v2.1.8 (Price et al., 2010), considering the Jones-Taylor-Thornton model of amino acid evolution (Jones et al., 1992). Furthermore, the Shimodaira-Hasegawa test (Shimodaira and Hasegawa, 1999) was used to check the reliability of each node. The visualization of the final topology was performed with the online tool iTOL v6 (Letunic and Bork, 2021), and the intersection of the shared orthologous proteins among the species under study was calculated with the R package “UpSetR” v1.4.0 (Conway et al., 2017).

### 2.5. Ecological distribution

With the aim to determine the presence and ecological distribution in hypersaline environments of strain 1BSP15-2V2^T^, as well as those of the previously described members of the genera *Aliifodinibius*, *Balneola*, *Fodinibius*, *Gracilimonas*, *Halalkalibaculum*, and *Rhodohalobacter*, a fragment recruitment analysis was performed against different environmental metagenomic datasets (Supplementary Table S2) showing similar metadata features to avoid bias due to them. The rRNA gene sequences of each of the analyzed genomes were masked due to the highly conserved nature of those genomic regions. Subsequently, the ≥30 bp high-quality metagenomic reads were BLASTn v2.2.28+ searched against each of the query genomes.

### 2.6. Fatty acids composition and phenotypic characterization

For the determination of the fatty acid profile, strain 1BSP15-2V2^T^ was grown on MA medium supplemented with 10% (w/v) NaCl at 37°C for 5 days. Fatty acids were determined by gas chromatography with an Agilent 6850 system at the Spanish Type Culture Collection (CECT), Valencia, Spain. The protocol recommended by the MIDI Microbial Identification System was followed (Sasser, 1990), using the TSBA6 library (MIDI, 2008).

Colonial morphology and pigmentation of strain 1BSP15-2V2^T^ were observed in the same SMM medium used for isolation supplemented with 7.5% (w/v) SW solution at pH 7.5, after 5 days of incubation at 37°C. Cell morphology and motility were examined by phase contrast microscopy (Olympus CX41). Anaerobic growth was determined after incubation on the above-mentioned medium, conditions, and time, using the AnaeroGen™ system (Oxoid).

The range and optimal salt concentration, pH, and temperature requirements for strain 1BSP15-2V2^T^ were determined by monitoring the optical density. Absorbance at 600 nm was measured every 2 h for 72 h using an Infinite M Nano (Tecan, Grödig, Austria) microplate reader. The incubation was set at 37°C with linear shaking. For determination of the salinity requirements, the SMM liquid medium was supplemented with SW stock to give a final salt concentration of 0 (in this case only distilled water was added), 3, 5, 6, 7.5, 9, 10, 12, 14, 15, 16, 17, 19, 20, 22, and 25% (w/v). To determine pH values supporting growth, the pH was adjusted to 3.0, 4.0, 5.0, 6.0, 7.0, 7.5, 8.0, 9.0, and 10.0 using a buffered system. Optimal temperature and range were ascertained in the same SMM liquid medium at the optimal salinity and pH values for the growth of strain 1BSP15-2V2^T^ at different temperatures: 4, 13, 14, 15, 20, 28, 34, 37, 40, 42, 43, 44, and 45°C. In this case, the optical density was measured in a Spectronic 20D+ (ThermoSpectronics, Cambridge, United Kingdom).

In order to phenotypically characterize the new isolate, strain 1BSP15-2V2^T^ was routinely grown in SMM medium supplemented with 7.5% (w/v) salts at pH 7.5 and 37°C for 5 days. However, the reference strains *A. halophilus* KCTC 42497^T^, *A. roseus* DSM 21986^T^, *A. salicampi* KACC 19060^T^, “*A. salipaludis*” KCTC 52855^T^, *A. saliphilus* KACC 19126^T^, and *A. sediminis* DSM 21194^T^ were routinely grown in the medium recommended by the culture collections, that is, R2A medium supplemented with 7.5% (w/v) SW solution at the same pH, temperature, and time than those for strain 1BSP15-2V2^T^. All biochemical tests were accomplished under these culture conditions. Catalase activity was examined by adding a 3% (w/v) H_2_O_2_ solution to colonies (Cowan and Steel, 1965), and oxidase activity was determined with 1% (v/v) tetramethyl-p-phenylenediamine (Kovacs, 1956). The following tests were determined as described by Cowan and Steel (1965): hydrolysis of gelatin, starch, Tween 80, DNA, casein, and aesculin, production of indole, methyl red and Voges–Proskauer tests, Simmons’ citrate, nitrate, and nitrite reduction, H_2_S production, urease, and phenylalanine deaminase. Acid production from carbohydrates was determined using a modified phenol red base medium prepared with 7.5% (w/v) salts supplemented with 0.05% (w/v) yeast extract and 1% (w/v) of the filter-sterilized carbohydrate to be checked (Cowan and Steel, 1965; Ventosa et al., 1982). The medium described by Koser (1923), as modified by Ventosa et al. (1982), was used to assess the utilization of different substrates as sole carbon and energy sources or as sole carbon, nitrogen, and energy sources. The studied carbohydrates, alcohols, organic acids, and amino acids were added as filter-sterilized solutions to give a final concentration of 1 g L^−1^ for all of them, except for carbohydrates, whose final concentration was 2 g L^−1^.

## 3. Results and discussion

### 3.1. Heavy metal contamination of the studied samples

To our best knowledge there are no reference studies on the concentration of heavy metals in the hypersaline soils from the Odiel Saltmarshes National Area, but it is known that the Odiel river waters and sediments contain high concentrations of arsenic, cadmium, copper, lead, and zinc due to unregulated industrial and mining activities in the past (Sainz et al., 2002, 2004). The sampled hypersaline soil presented a pH value of 8.4 and an electrical conductivity of 24.57 mS/cm. The concentrations of arsenic, cadmium, copper, lead, and zinc were (mg kg^−1^): 14.86, 0.47, 106.40, 24.81, and 129.30, respectively. Arsenic, copper, and zinc presented values above the reference ranges for non-contaminated soils, according to the criteria of the Government of the region of Andalucía, where the Odiel Saltmarshes National Area is located (Consejería de Medio Ambiente, 1999), while cadmium and lead concentrations were within the reference ranges (Table 1).

**Table 1:** Concentration of the most recurring heavy metal contaminants in the sampling area.

Heavy metal	Sample concentration (mg kg^−1^)	Reference range for non-contaminated soils (mg kg^−1^)
Arsenic	14.86	2–5
Cadmium	0.47	0.4–0.8
Copper	106.40	17–100
Lead	24.81	10–50
Zinc	129.30	10–70

The reference ranges refer to soils categorized as not contaminated in Andalucía (Spain), where the Odiel Saltmarshes National Area is located.

### 3.2. The new isolated strain constitutes a novel species of the genus *Fodinibius*

**Figure 1:**
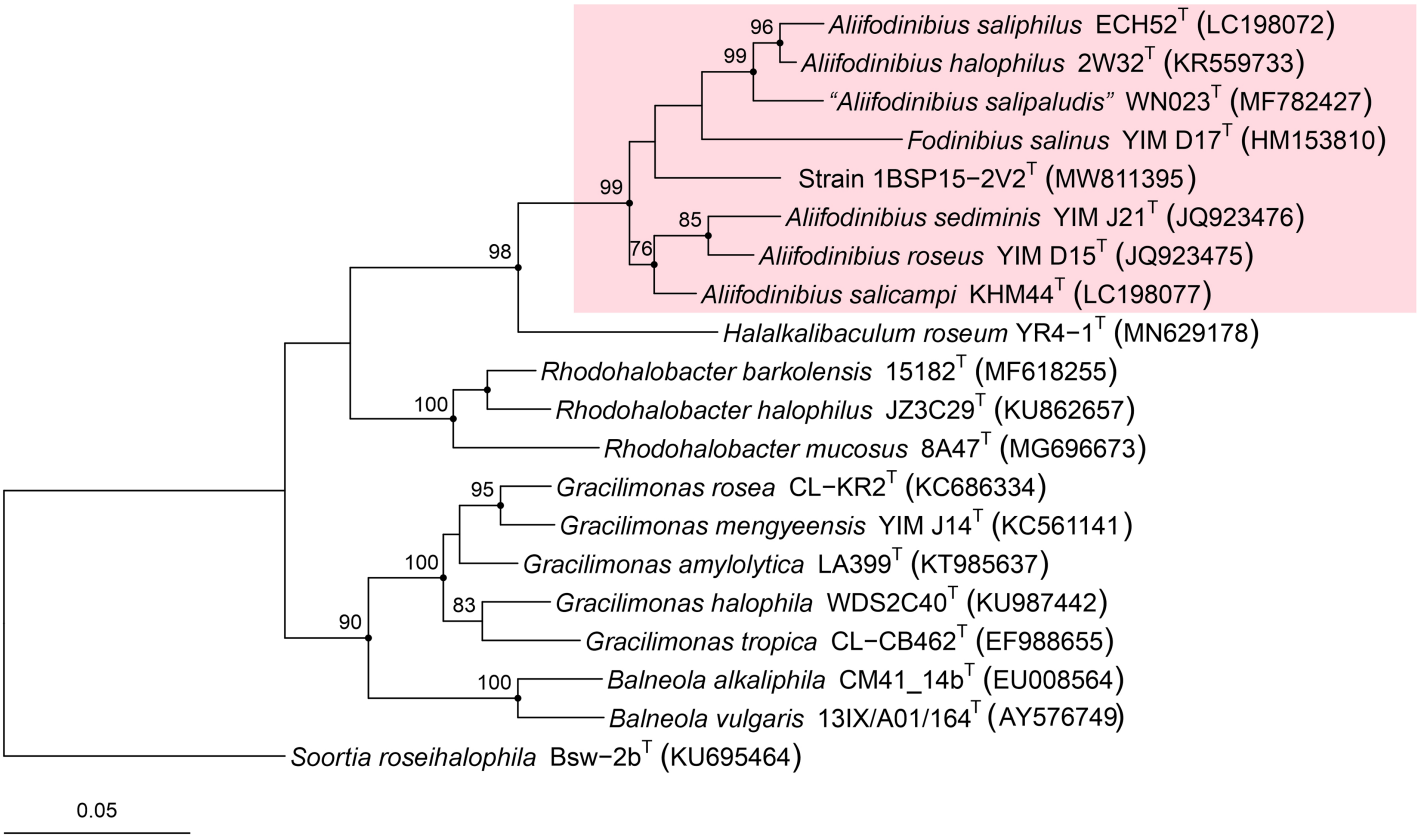
Maximum-likelihood phylogenetic tree based on the comparison of the 16S rRNA gene sequences showing the relationships among members of the family *Balneolaceae*. Bootstrap values equal or higher than 70% based on 1,000 pseudoreplicates are indicated at selected branch nodes. Filled circles indicate that the corresponding node was also obtained in the trees generated with the maximum-parsimony and neighbor-joining algorithms. The species *Soortia roseihalophila*, the closest relative to the family *Balneolaceae*, was selected as an outgroup. Bar, 0.05 substitutions per nucleotide position.

The strain 1BSP15-2V2^T^ was cultured in pure culture after an extensive screening of the prokaryotic diversity carried out in hypersaline soil samples obtained from the Odiel Saltmarshes Natural Area (Huelva, Southwest Spain), where more than 4,000 isolates were investigated. The 1,455 bp-long 16S rRNA gene sequence of this strain showed the closest relationship with *A. roseus* DSM 21986^T^ and *A. saliphilus* ECH52^T^ (95.02% sequence identity, in both cases). Reconstruction of the 16S rRNA gene-based phylogeny by the maximum-likelihood algorithm clustered strain 1BSP15-2V2^T^ together to the species of the genus *Aliifodinibius* (Figure 1). The species *Fodinibius salinus* YIM D15^T^ was also clustered within the other species of the genus *Aliifodinibius*, which suggests that the two genera are closely related. However, the bootstrap value for the node harboring strain 1BSP15-2V2^T^ is below 70% and the topology differs sightly depending on the algorithms used (maximum-likelihood, maximum-parsimony, or neighbor-joining). A similar issue can be observed for the branch that accommodates *Fodinibius salinus* YIM D15^T^. Therefore, the robustness of this tree is not strong enough as to infer reliable relationship between the species of the genera *Aliifodinibius* and *Fodinibius,* and strain 1BSP15-2V2^T^, but offers an approach to their evolutionary history.

**Figure 2:**
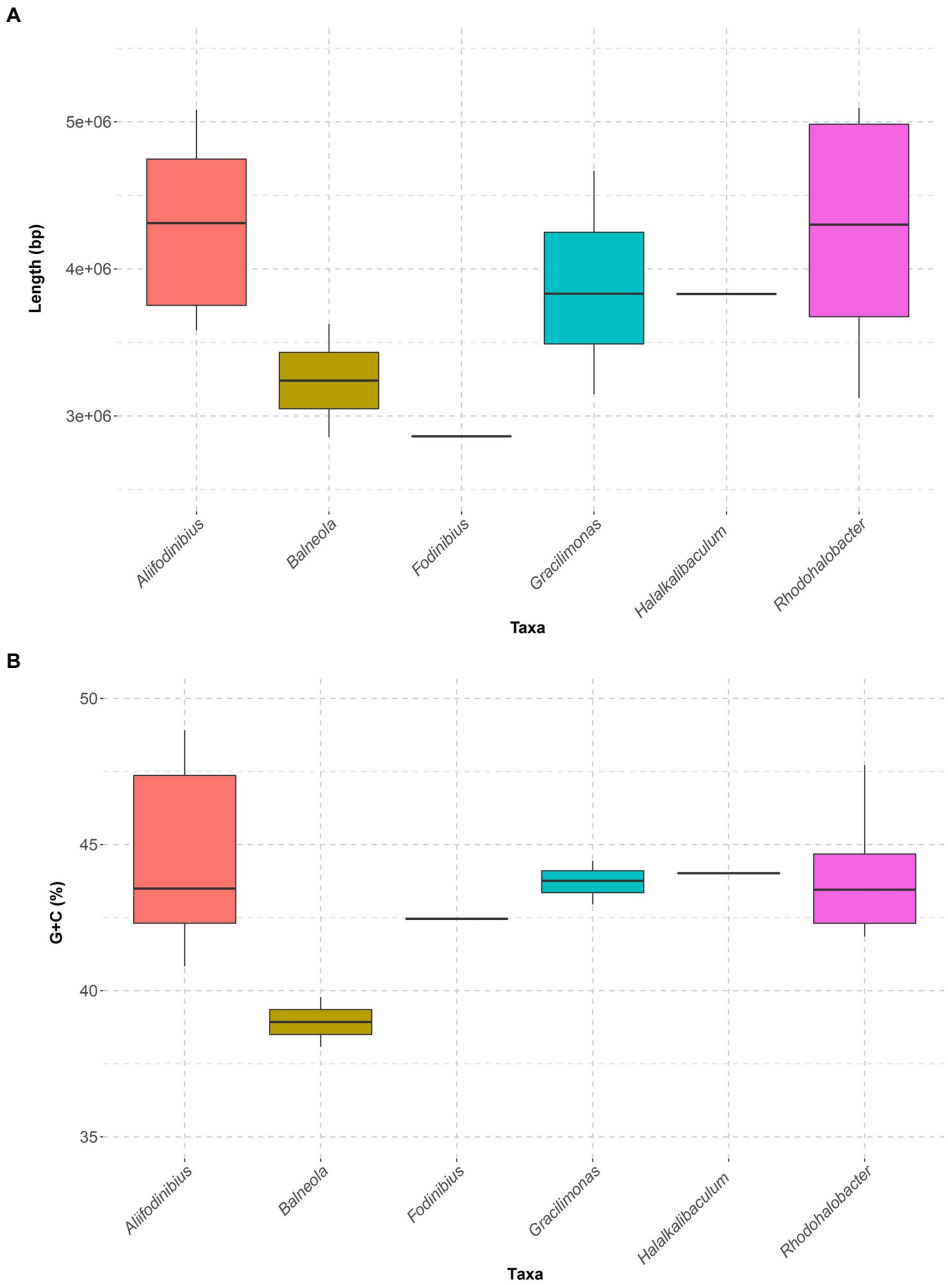
Boxplots summarizing genome size **(A)** and G + C content **(B)** of the genera of the family *Balneolaceae*, sorted alphabetically. The family *Balneolaceae* includes genera with very different genomes sizes, but their G + C percentages are mostly stable around 43 mol%, except for the type genus *Balneola*, below 40 mol%.

The draft genome sequence of strain 1BSP15-2V2^T^ (GCF_026229185.1) was assembled into 43 contigs, with a total size of 4,850,852 bp, and a DNA G + C content of 44.47 mol%. We also obtained the genome sequence of the closely related species *A. salicampi* KACC 19060^T^ (GCF_026228885.1), not previously available in public databases, which contains 13 contigs, slightly shorter size (3,937,193 bp), and a genomic G + C content of 42.75 mol%. The members of the genus *Aliifodinibius* possess genome sizes ranging from 3,583,276 bp (“*A. salipaludis*” WN023^T^) to 5,082,244 bp (*A. roseus* DSM 21986^T^) (Figure 2A; Supplementary Table S1). *Fodinibius salinus* DSM 21935^T^ has one of the smallest genomes of the members within the family *Balneolaceae* (2,861,751 bp), but with similar G + C percentage than those of the genera *Aliifodinibius*, *Gracilimonas*, *Halalkalibaculum*, and *Rhodohalobacter*. On the contrary, the species of the genus *Balneola*, the type genus of the family *Balneolaceae*, have smaller average genome size than the rest of members of the family (except the genus *Fodinibius*), as well as the lowest G + C content (<40 mol%) (Figures 2A,B; Supplementary Table S1).

**Figure 3:**
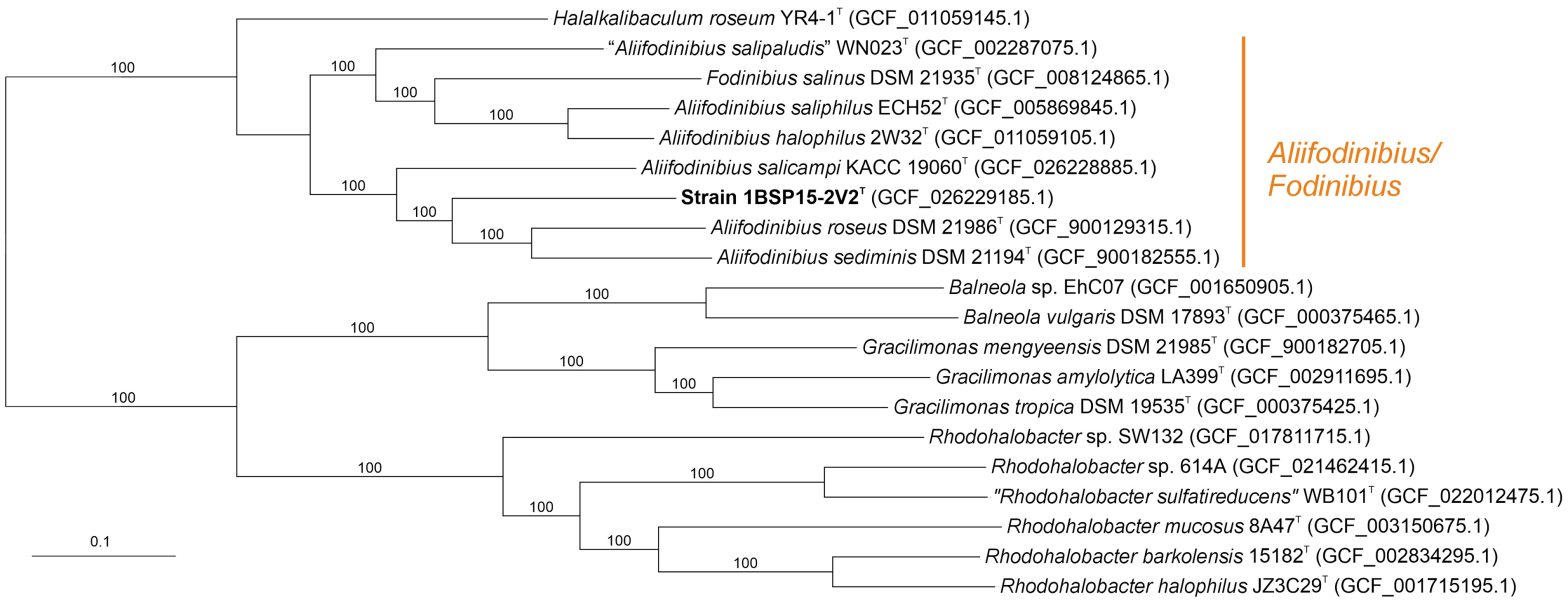
Approximately maximum-likelihood phylogenomic tree based on 1,049 concatenated core protein sequences of members of the family *Balneolaceae*. Bootstrap values ≥70% are indicated above the branches. Bar, 0.1 substitutions per nucleotide position.

The approximated maximum-likelihood phylogenomic tree, based on the concatenation of 1,049 translated core genes, showed that the strain 1BSP15-2V2^T^ clustered again with the species of the genus *Aliifodinibius* (Figure 3). This clustering was supported by a 100% bootstrap value, indicating the robustness of the group, which was unclear in the 16S rRNA gene-based phylogeny (Figure 1). Furthermore, *Fodinibius salinus* DSM 21935^T^, the only described species of this genus, also clustered with 100% branch support together to the species of the genus *Aliifodinibius*, rather than forming a separated and independent branch. This fact seems to indicate that members of the genera *Aliifodinibius* and *Fodinibius* could be actually merged into the single genus *Fodinibius*.

**Figure 4:**
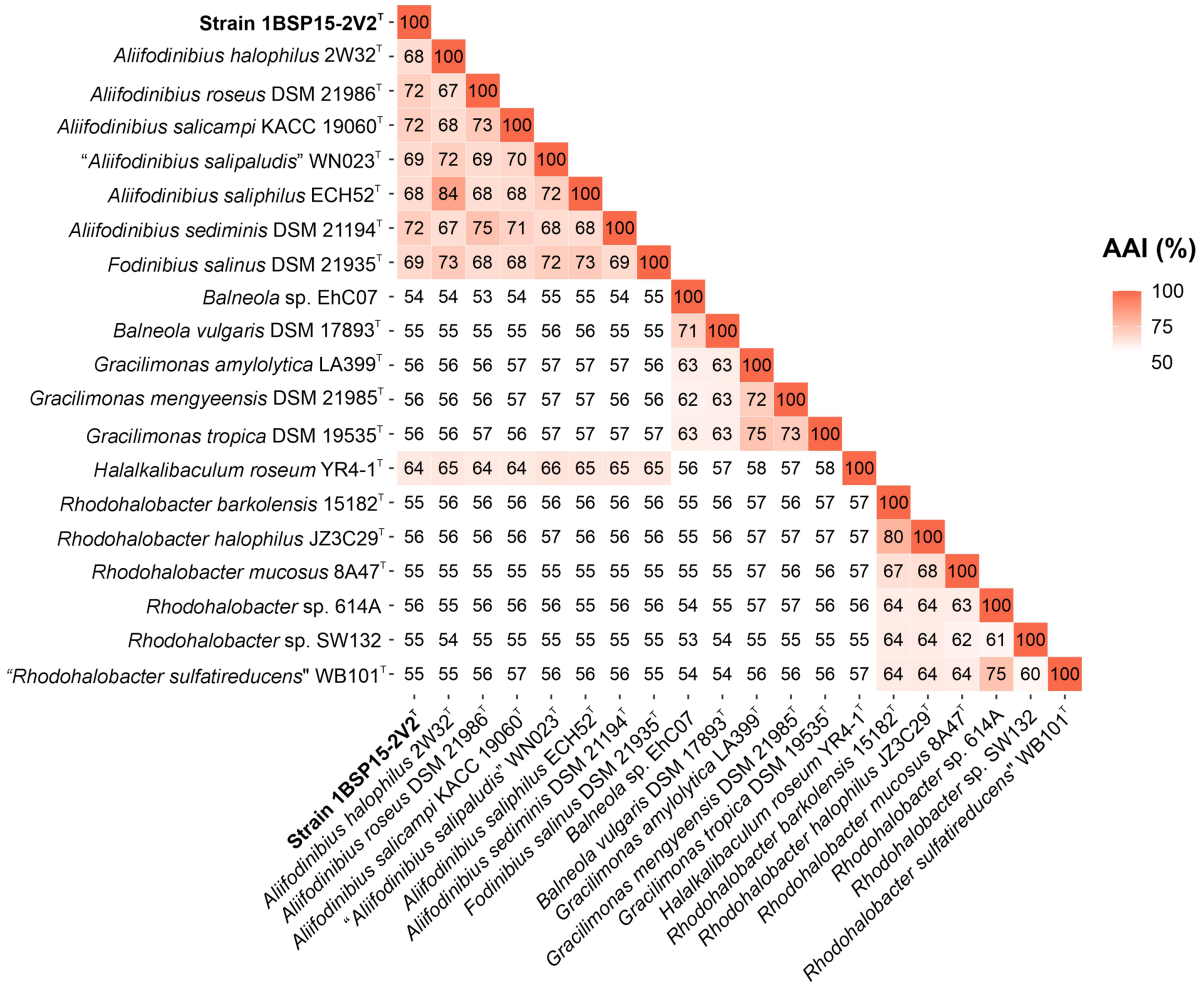
AAI matrix among the 20 studied genomes of the family *Balneolaceae*. AAI values among species of the cluster *Fodinibius*-*Aliifodinibius* were all higher than the threshold (65%) accepted for genus delineation. Isolate 1BSP15-2V2^T^ shared values above 68% with species of the genera *Aliifodinibius* and *Fodinibius*, and equal or lower than 64% with other members of the family *Balneolaceae*, hinting at the affiliation of the novel strain to the genera *Aliifodinibius*/*Fodinibius*.

**Figure 5:**
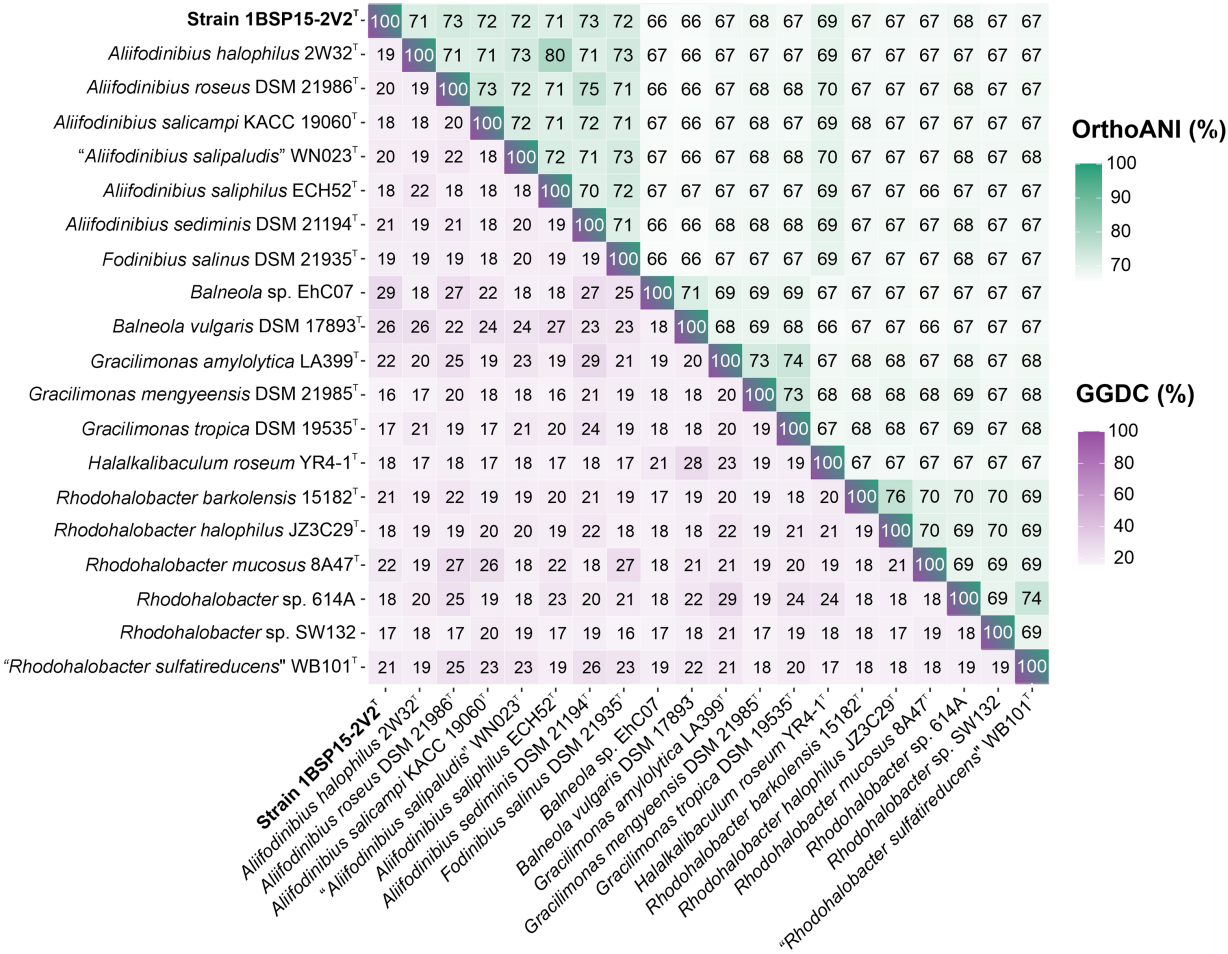
OrthoANI (upper triangle) and GGDC (lower triangle) matrix among the 20 studied genomes of the family *Balneolaceae*. All GGDC (purple) and orthoANI (green) values were below the cutoff for species delineation, indicating that all the genomes belong to different species.

Concerning the OGRIs analysis, AAI values between strain 1BSP15-2V2^T^ and the other members of the genus *Aliifodinibius* ranged from 72.1% (with *A. sediminis* DSM 21194^T^) to 68.1% (with *A. halophilus* 2W32^T^) (Figure 4). These values were above the 65–72% threshold suggested to circumscribe members of the same genus (Konstantinidis and Tiedje, 2007; Konstantinidis et al., 2017). Similar AAI values were obtained for *Fodinibius salinus* DSM 21935^T^, which varied from 72.9% (with *A. halophilus* 2W32^T^) to 68.1% (with *A. roseus* DSM 21986^T^) with respect to the species of the genus *Aliifodinibius*, but were equal or lower than 64.9% for *Halalkalibaculum roseum* YR4-1^T^ and the remaining species of the genera of the family *Balneolaceae* (Figure 4). Furthermore, orthoANI percentages among the analyzed genomes within the *Balneolaceae* were all below the 95% threshold established for species differentiation (Goris et al., 2007; Richter and Rosselló-Móra, 2009; Chun and Rainey, 2014), including strain 1BSP15-2V2^T^, which shares the highest similarity with *A. roseus* DSM 21986^T^ and *A. sediminis* DSM 21194^T^ (72.7% for both species) (Figure 5). These data undoubtedly indicate that all strains under study represented different species. The same conclusion can be inferred from dDDH results, where all values are far below the 70% cutoff defined for species delineation (Stackebrandt and Goebel, 1994; Auch et al., 2010) (Figure 5).

**Figure 6:**
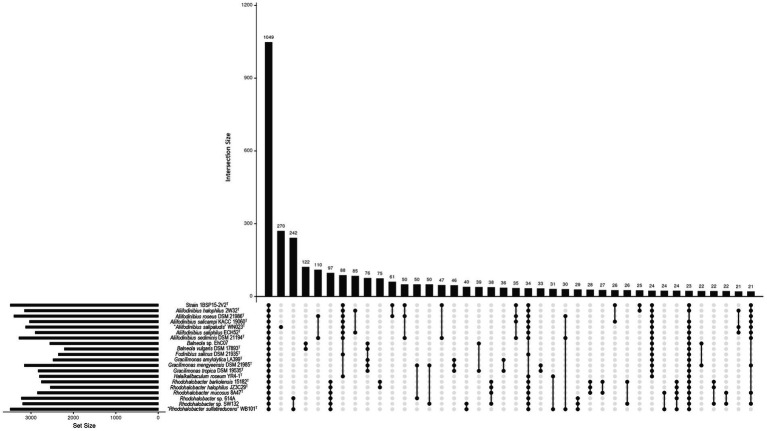
Upset plot showing the proteins shared among the 20 *Balneolaceae* genomes under study. Only the 40 most frequent intersecting sets are displayed. From a pan genome of 8,113 translated genes, 1,049 were encoded in all the genomes (core genome). “*Aliifodinibius salipaludis*” WN023^T^ harbored the highest number of unique translated genes (270). The closely related genera *Aliifodinibius*, *Fodinibius*, and *Halalkalibaculum* shared 88 translated genes.

The distribution of core, soft-core, and accessory genomic content among the 20 genomes of the family *Balneolaceae* investigated here are shown in Figure 6. All the studied genomes of the species of the family *Balneolaceae* share a total of 1,049 core genes. The species “*Aliifodinibius salipaludis*” WN023^T^ possesses the highest number of unique genes (270), although it has the smaller genome size (3.58 Mb) and the lower number of CDS (3,143) (Supplementary Table S1). This could be related with the habitat where “*Aliifodinibius salipaludis*” WN023^T^ dwells, since to date it is the only member of the family isolated from saline-alkaline soil instead of hypersaline sediments or salterns (Zhao et al., 2020). However, our new strain 1BSP15-2V2^T^ was also isolated from saline soils and its exclusive genetic material is not high enough (12 unique genes) as to be displayed among the most frequent intersections (Figure 6). Strain 1BSP15-2V2^T^ shares a total of 61 genes exclusively with the type species of the genus *Aliifodinibius, A. roseus* DSM 21986^T^, and 50 additional genes if the species *A. sediminis* DSM 21194^T^ is also considered. In any case, strain 1BSP15-2V2^T^ shares a higher number of genes with the species of the genus *Aliifodinibius* than with any other of the remaining genera of the family *Balneolaceae*. Moreover, a large cluster of 88 genes that are present solely within the genera *Aliifodinibus* and *Fodinibius*, as well as the genus *Halalkalibaculum*, points to their closed evolutionary relationships (Figure 6). Similarly, 97 genes are exclusively shared between the members of the genus *Rhodohalobacter*. In summary, we observed that the most closely related the species are the highest number of genes they share. Further differences in the protein functions are described in Section 3.4.

### 3.3. Chemotaxonomic and phenotypic characterization support the placement of the new isolate within the genus *Fodinibius*

The major fatty acids determined for strain 1BSP15-2V2^T^ were iso-C_15:0_ (36.4%), C_16:1_ *ω*7c and/or C_16:1_ *ω*6c (24.7%), and iso-C_17:1_ *ω*9c and/or 10-methyl C_16:0_ (10.8%). The profile of this strain is similar to those of the type strains of the species of the genera *Aliifodinibius* and *Fodinibius* (Supplementary Table S3). No clear differential profiles were obtained for species of the genera *Aliifodinibius* and *Fodinibius*, supporting the hypothesis that they all belong to the same genus.

The strain 1BSP15-2V2^T^ formed irregular, opaque, and orange-red pigmented colonies. Cells were Gram-stain-negative, non-motile, non-endospore-forming rods, with a size of 0.05–0.10 × 0.20–0.30 μm. The detailed phenotypic characteristics of strain 1BSP15-2V2^T^ are included in the new species description. Besides, additional phenotypic data for the previously described species of the genera *Aliifodinibius* and *Fodinibius* included in this study are shown in Supplementary Table S4. The morphological and many of the biochemical characteristics of the isolate 1BSP15-2V2^T^ agree with those reported for the other members of the genera *Aliifodinibius* and *Fodinibius*, and thus, support the placement of strain 1BSP15-2V2^T^ within the genus *Fodinibius* (which should also include the described species of *Aliifodinibius*). Besides, strain 1BSP15-2V2^T^ possesses several features that allow to distinguish it from the close-related species of the genera *Aliifodinibius* and *Fodinibius*, namely, the ability to use several amino acids as sole carbon, nitrogen, and energy sources (Supplementary Table S4).

### 3.4. Functional genomic analysis reveals the potential ecological role of the new strain as a biotin producer

**Figure 7:**
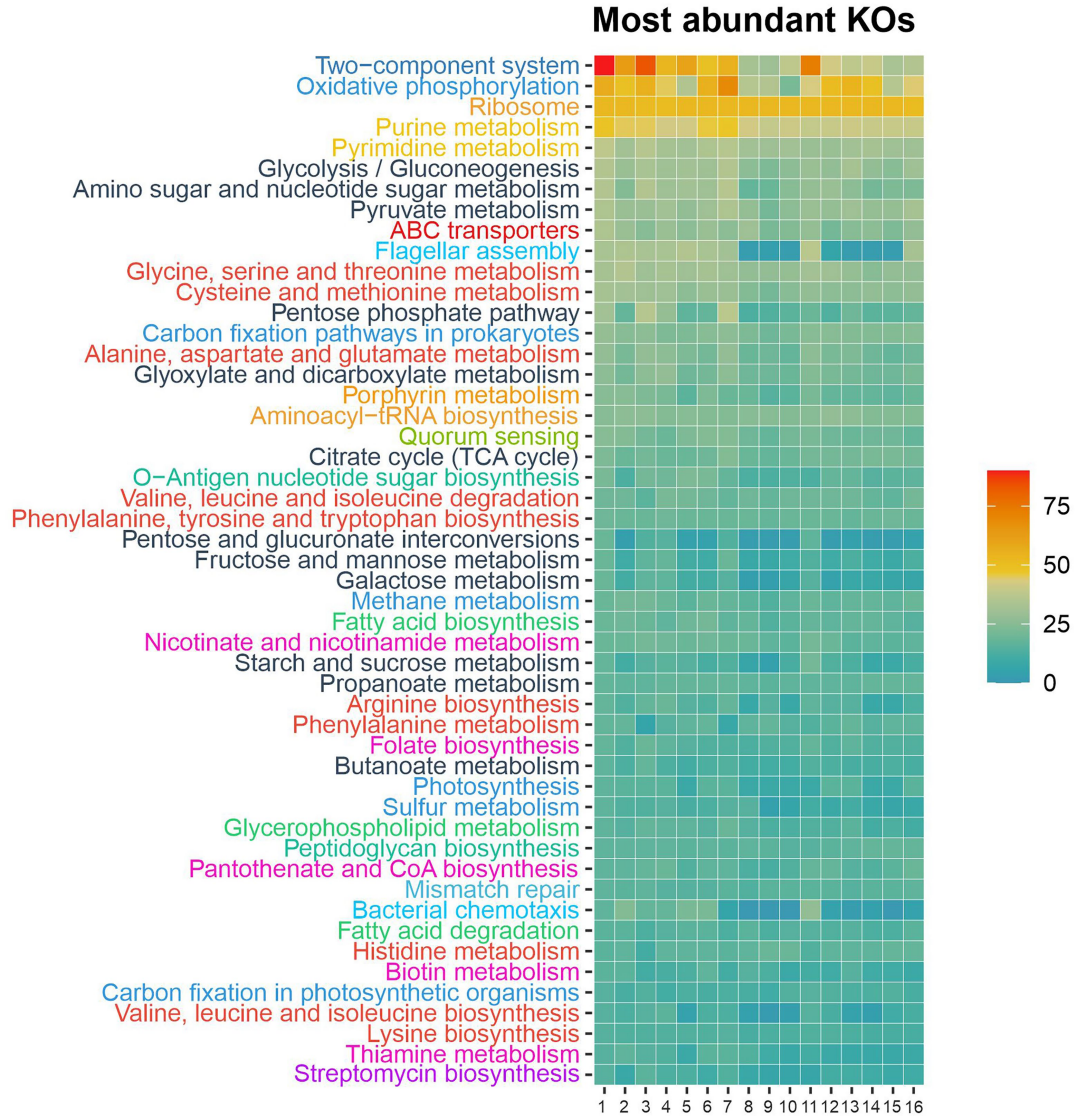
KO numbers grouped by KEGG categories annotated for the representative genomes of the family *Balneolaceae*. KEGG pathways are sorted according to their abundance in the strain 1BSP15-2V2^T^ and only the 50 most abundant are displayed. The most prominent pathways detected in those genomes belonged the following KEGG global categories: signal transduction (dark blue), energy metabolism (blue), translation (orange), nucleotide metabolism (yellow), carbohydrate metabolism (black), membrane transport (dark red), cell motility (light blue), amino acid metabolism (light red), cellular community (light green), glycan biosynthesis and metabolism (dark green), lipid metabolism (green), metabolism of cofactors and vitamins (pink), and biosynthesis of other secondary metabolites (purple). 1. Strain 1BSP15-2V2^T^; 2. *Aliifodinibius halophilus* 2W32^T^; 3. *Aliifodinibius roseus* DSM 21986^T^; 4. *Aliifodinibius salicampi* KACC 19060^T^; 5. “*Aliifodinibius salipaludis*” WN023^T^; 6. *Aliifodinibius saliphilus* ECH52^T^; 7. *Aliifodinibius sediminis* DSM 21194^T^; 8. *Fodinibius salinus* DSM 21935^T^; 9. *Balneola vulgaris* DSM 17893^T^; 10. *Gracilimonas amylolytica* LA399^T^; 11. *Gracilimonas mengyeensis* DSM 21985^T^; 12. *Gracilimonas tropica* DSM 19535^T^; 13. *Halalkalibaculum roseum* YR4-1^T^; 14. *Rhodohalobacter barkolensis* 15182^T^; 15. *Rhodohalobacter halophilus* JZ3C29^T^; 16. *Rhodohalobacter mucosus* 8A47^T^.

BlastKOALA annotation identified a total of 1,986 different KO numbers across the 16 available genomes of the type strains of species of the family *Balneolaceae*. Those KO numbers were classified according to KEGG pathway database, and the 50 most abundant pathways are shown in Figure 7. Strain 1BSP15-2V2^T^ seems to exhibit a functional profile like that of the already described species of the genera *Aliifodinibius*/*Fodinibius* and the rest of the genera within the family *Balneolaceae*. Nevertheless, it must be noted that 22 KO numbers were only present in strain 1BSP15-2V2^T^ and not in any other member of the family. Among those unique functional orthologs, we detected *frdA*, *frdB*, *frdC*, and *frdD* genes in the genome of strain 1BSP15-2V2^T^, involved in the conversion of fumarate to succinate (Supplementary Table S5).

The most prevalent pathways of strain 1BSP15-2V2^T^ were included into energy, carbohydrate, and amino acid metabolism KEGG global categories. All the species of the genera *Aliifodinibius* and *Fodinibius* harbored genes involved in pyruvate metabolism, including the pyruvate oxidation pathway comprising KO numbers K00161 (*pdhA*, pyruvate dehydrogenase E1 component α-subunit), K00162 (*pdhB*, pyruvate dehydrogenase E1 component β-subunit), K00627 [*pdhC*, pyruvate dehydrogenase E2 component (dihydrolipoamide acetyltransferase)], and K00382 (*pdhD*, dihydrolipoamide dehydrogenase) (Supplementary Table S5). However, only strain 1BSP15-2V2^T^, *A. halophilus* KCTC 42497^T^, and *A. roseus* DSM 21986^T^ were able to use pyruvate as sole carbon and energy source when tested experimentally (Supplementary Table S4). The other genera of the family *Balneolaceae* showed the same *in-silico* metabolism for pyruvate, but it was not confirmed in this study under laboratory conditions.

Strain 1BSP15-2V2^T^ and the species of the genus *Aliifodinibius* possessed genes linked to flagellar assembly (Figure 7; Supplementary Table S5), in contrast to the genera *Fodinibius*, *Balneola*, *Halalkalibaculum*, and *Rhodohalobacter*, as well as the species *Gracilimonas amylolytica* LA399^T^ and *G. tropica* DSM 19535^T^, although the route was incomplete in all the cases. This finding agrees with the fact that none of the descriptions of the species of the genus *Aliifodinibius* reported the cellular motility (Wang et al., 2013; Xia et al., 2016; Cho et al., 2017; Cho and Whang, 2020; Zhao et al., 2020). Cells of 1BSP15-2V2^T^ did not show motility either in the present study. The presence of a broken pathway for flagellar motility seems to indicate that the ancestor of the family *Balneolaceae* may have had that feature, which was gradually lost as it was not required for their survival in the nature. Major differences between the functional profile of species of the genera *Aliifodinibius* and *Fodinibius* are related to bacterial motility, that is flagellar assembly and flagellar motor switch (two component system). This supports the theory proposed above about the loss of motility-related genes, which might have happened quicker in *F. salinus*. Other than those two pathways, *F. salinus* presents a mostly similar profile to the species of the genus *Aliifodinibius*. We can find minor differences between the genomes of the studied species of *Aliifodinibius/Fodinibius*, such as less abundance of functions related to oxidative phosphorylation and purine metabolism in the species “*Aliifodinibius salipaludis*” and *F. salinus*, though similar between them, and an oscillation in the number of genes encoding enzimatic activities in the pentose phosphate pathway. In any case, all the studied genomes are uncompleted, and their range size is broad among the members of the *Aliifodinibius/Fodinibius*. Hence, we can only speculate about the presence or lack of functions, as it may be due to a deficient annotation or assembly with the current bioinformatic tools, or to an adaptation of the bacteria to their particular environment.

**Figure 8:**
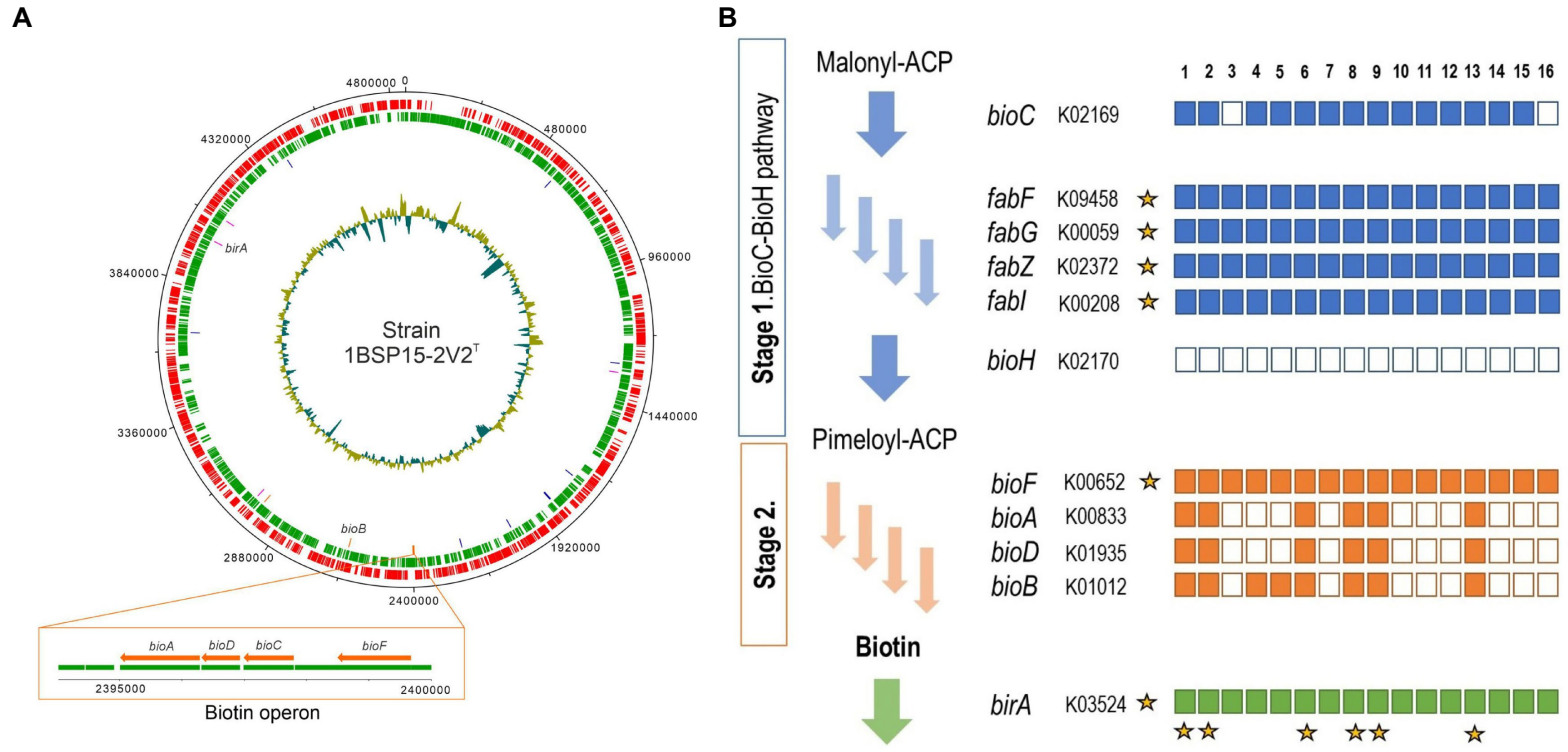
**(A)** Circular genome map of strain 1BSP15-2V2^T^ annotated by Prokka. The graph was generated with “DNAPlotter” (Carver et al., 2009). Circles indicate, from outside inwards: coding sequences in the leading strand (red); coding sequences in the lagging strand (green); coding sequences related to the biotin biosynthesis; G + C content. Biotin pathway-related genes are displayed in colors: stage 1 of biosynthesis, blue; stage 2 of biosynthesis, orange; regulation and transport, pink. A diagram of the biotin operon arrangement is shown in the box. Genes *bioA*, *bioD*, *bioC*, and *bioF* are located in the same region of the genome with a hypothetical protein encoded between *bioF* and the rest of the *bioADC* genes. The organization in form of operon allows the cotranscript of their proteins in the correct stoichiometry (Sirithanakorn and Cronan, 2021). *bioB* and *birA* genes are placed in a different region of the chromosome. **(B)** Biotin biosynthesis pathway for members of the family *Balneolaceae*. Although almost the complete BioC-BioH pathway was detected in most of the genomes, *bioH* gene was missing for all of them. Stars indicate either that the gene is present in all the genomes or that the pathway (except *bioH* gene) is complete for the corresponding genome. 1. Strain 1BSP15-2V2^T^; 2. *Aliifodinibius halophilus* 2W32^T^; 3. *Aliifodinibius roseus* DSM 21986^T^; 4. *Aliifodinibius salicampi* KACC 19060^T^; 5. “*Aliifodinibius salipaludis*” WN023^T^; 6. *Aliifodinibius saliphilus* ECH52^T^; 7. *Aliifodinibius sediminis* DSM 21194^T^; 8. *Fodinibius salinus* DSM 21935^T^; 9. *Balneola vulgaris* DSM 17893^T^; 10. *Gracilimonas amylolytica* LA399^T^; 11. *Gracilimonas mengyeensis* DSM 21985^T^; 12. *Gracilimonas tropica* DSM 19535^T^; 13. *Halalkalibaculum roseum* YR4-1^T^; 14. *Rhodohalobacter barkolensis* 15182^T^; 15. *Rhodohalobacter halophilus* JZ3C29^T^; 16. *Rhodohalobacter mucosus* 8A47^T^.

The biosynthesis pathway for biotin (vitamin B_7_) appears to be present in the species of the family *Balneolaceae*. Though the understanding of biotin biosynthesis is still fragmentary, previous studies (Cronan, 2018; Manandhar and Cronan, 2018) propose two stages: the synthesis of a pimelate moiety, and the assembly of the bicyclic rings of the biotin molecule. The latter is mostly conserved, with four associated genes: *bioF*, *bioA*, *bioD*, and *bioB*, all of them present in strain 1BSP15-2V2^T^ (Figure 8A; Supplementary Table S5). These genes were also detected in *A. halophilus*, *A. saliphilus*, *Fodinibius salinus, Halalkalibaculum roseum*, and *Balneola vulgaris* (Figure 8B; Supplementary Table S5). Only *bioB* and *bioF* were present in *A. salicampi* and “*A. salipaludis*.” However, the *bioF* gene, which performs the first step of the assembly of the bicyclic rings, can be found in all the studied species of the family *Balneolaceae*. On the contrary, there are at least three known pathways to accomplish the first stage of biotin biosynthesis: the BioC-BioH and the BioI-BioW pathways, studied in *Escherichia coli* and *Bacillus subtilis*, respectively (Lin and Cronan, 2011), and the most recently proposed BioZ pathway (Zhang et al., 2021). However, the BioC-BioH pathway seems to be the most prevalent of these three (Feng et al., 2014; Wei et al., 2019). Genes *bioC*, *fabF*, *fabG*, *fabZ*, and *fabI* from the BioC-BioH pathway are found in all the species of the genus *Aliifodinibius,* except the *bioC* gene lacking in *A. roseus*. These genes are also present in strain 1BSP15-2V2^T^ and *Fodinibius salinus*, as well as in most species of the family *Balneolaceae*. Noteworthy, all studied genomes of the family seem to lack the *bioH* gene, which performs the last step of the BioC-BioH pathway. However, homologous genes have been found in other organisms as replacements (Rodionov et al., 2002), such as *bioG* in *Haemophilus* sp. (Shi et al., 2016), *bioK* in *Synechococcus* sp. (Shapiro et al., 2012), *bioJ* in *Francisella* sp. (Wei et al., 2019), and *bioV* in *Helicobacter* sp. (Bi et al., 2016). None of these alternative genes were found in the genomes under study, but we cannot discard the possibility that the family *Balneolaceae* harbors a different homologous gene that hydrolyzes the pimeloyl-ACP methyl ester into pimeloyl-ACP in the last step of the BioC-BioH pathway (Lin and Cronan, 2011). On the other hand, all the species of the family *Balneolaceae* present *birA, accB*, and *accC* genes in their genomes. The protein BirA has a dual function: attach biotin to AccB protein and down-regulate biotin biosynthesis in the absence of free AccB acceptor protein. AccB is a carrier protein that forms a complex with the AccC subunit (Choi-Rhee and Cronan, 2003), so both macromolecules indirectly regulate the transcription of the biotin operon. Low levels of available AccB protein, that is when AccB protein is biotinylated or unbiotinylated but bonded to AccC, avoid biotin transfer from BirA to AccB, resulting in higher levels of BirA ligand bound to biotin. The accumulation of BirA-bound biotin represses biotin operon transcription (Abdel-Hamid and Cronan, 2007). In summary, the genome of 1BSP15-2V2^T^, as well as those of the rest of the species of the family *Balneolaceae*, contain genes involved in the regulation of biotin biosynthesis. With a single exception, they also have the complete set of *bioC*, *fabF*, *fabG*, *fabZ*, and *fabI* genes that catalize the first steps of biotin biosynthesis, albeit all of them lack the *bioH* gene, which encodes the last enzyme of this first stage. Since several *bioH* homologs have been reported in the literature, the species of the family *Balneolaceae* are hypothesized to harbor one of those not-yet described homologous genes. Considering that the more conserved second stage (*bioF*, *bioA*, *bioD*, and *bioB* genes) is only present in the genomes of strain 1BSP15-2V2^T^ and other five members of the family, the complete (or almost complete) biotin biosynthesis pathway was only identified in the genomes of strain 1BSP15-2V2^T^, *A. halophilus*, *A. saliphilus*, *Fodinibius salinus*, *Halalkalibaculum roseum*, and the type species of the family, *Balneola vulgaris*, whereas it was missing from the genera *Gracilimonas* and *Rhodohalobacter*. Since the biotin biosynthetic pathway is well conserved across biotin producers (Lin and Cronan, 2011), it may be explained the presence of at least some of those genes within all described species of the family *Balneolaceae*, even if the pathway is uncompleted. Biotin plays an important role in fatty acid synthesis, amino acid metabolism, and gluconeogenesis in bacteria, archaea, and eukaryotes (Knowles, 1989; Jitrapakdee and Wallace, 2003). The biosynthesis of biotin is exclusive of some prokaryotes, fungi, and plants but it is an essential cofactor for mammals too, which depend on the exogenous supply from the diet or the microbiota (Cronan et al., 2014). For example, vitamin B_7_ deficiency in humans is associated with neurological diseases (León-Del-Río, 2019). It is a high-costing metabolic pathway, especially the steps carried out by the proteins coded by *bioC*, *bioA* and *bioB* genes. Hence, most organisms prefer an exogenous uptake of this cofactor (Sirithanakorn and Cronan, 2021). The isolated species could play a role in its ecosystem by suppling biotin to those organisms that lack the mechanisms for *de novo* synthesis. Besides, it might be further explored as an interesting source of biotin for human deficiency with biotechnological application. Nowadays, biotin for pharmacological purposes is chemically synthetize since bacterial biosynthesis has given unsuccessful results to date (Sirithanakorn and Cronan, 2021). This is, partly, due to the uncomplete understanding of the biotin pathway in prokaryotes. Thus, the genomic identification of this route in strain 1BSP15-2V2^T^ will allow its targeting as a possible candidate for further studies of the biotin biosynthesis.

In addition to the biotin biosynthesis, strain 1BSP15-2V2^T^ also presents functional orthologs related to other cofactors and vitamins, such as folate, pantothenate and CoA biosynthesis, and metabolism of thiamine, nicotinate, and nicotinamide (Figure 7; Supplementary Table S5). On the other hand, only the species of the genera *Aliifodinibius* and *Fodinibius*, including the strain 1BSP15-2V2^T^, showed genes involved in the cobalamin (vitamin B_12_) transport system (*btuC*, *btuD*, and *btuF*), and all members of the family *Balneolaceae* harbors *btuB,* a vitamin B_12_ transporter. Moreover, a previous study has reported the presence of cobalamin-dependent enzymes in the metagenomic dataset SMO2, obtained from hypersaline soils at the Odiel Saltmarshes National Area (Durán-Viseras et al., 2019). These enzymes corresponded to the KO numbers K00525 (*nrdA*, ribonucleoside-diphosphate reductase α-chain), K01848 (*mcmA1*, methylmalonyl-CoA mutase, N-terminal domain), and K00548 (*metH*, 5-methyltetrahydrofolate–homocysteine methyltransferase). Two of them, K00525 and K00548, along with K01847 (*MUT*, methylmalonyl-CoA mutase) and K11942 (*icmF*, isobutyryl-CoA mutase), were found in the genomes of strain 1BSP15-2V2^T^ and members of the family *Balneolaceae*. However, none of them encoded the cobalamin biosynthesis pathway, which is not surprising, as it is a high-cost metabolic mechanism (Morris, 2012; Doxey et al., 2015). Thus, it seems to indicate that strain 1BSP15-2V2^T^ as well as the rest of the family *Balneolaceae* depend on an external source of vitamin B_12_, such as species of the haloarchaeal genera *Halobacterium* (Woodson et al., 2003) and *Halonotius* (Durán-Viseras et al., 2019).

### 3.5. Metalloresistant abilities to bear up against contaminated soils

As stated above, cadmium and lead concentrations in our sampled hypersaline soils were within the reference ranges for a non-contaminated soil, whereas arsenic, copper, and zinc concentrations were above the maximum thresholds (Table 1). Therefore, we can classify our samples as arsenic, copper, and zinc contaminated soils.

Several genes related to heavy metal resistance were found in the genome of strain 1BSP15-2V2^T^. The *ars* operon (KO numbers K03325, K03741, and K03892) (Supplementary Table S5) is responsible for reduction of arsenate, As (V), to arsenite, As (III), and its excretion from the cell (Chauhan et al., 2019; Islam et al., 2022). Arsenic resistance is believed to be a widespread mechanism among prokaryotes due to the high concentration of this metalloid in the early period of the Earth (Chen et al., 2020). On the other hand, the CzcCBA efflux system (K15725, K15726, K15727, and K16264) (Supplementary Table S5) provides resistance to cadmium, copper, and zinc (Legatzki et al., 2003). This pump has been found in many metallotolerant Gram-negative bacteria (Manara et al., 2012; Khan et al., 2015; Alquethamy et al., 2021). Furthermore, the *zntA* gene (K01534) (Supplementary Table S5) for Zn^2+^/Cd^2+^-exporting (Rensing et al., 1997; Noll and Lutsenko, 2000) was also detected in the genome of strain 1BSP15-2V2^T^. Previous studies have demonstrated the ability of CzcCBA efflux system to efficiently remove zinc and cadmium from the cytoplasm (Legatzki et al., 2003). Thus, the existence of an additional *zntA* gene-encoding pump in strain 1BSP15-2V2^T^ seems to be an adaptative mechanism to cope with the harsh metal conditions of the Odiel Saltmarshes National Area hypersaline soils. Nevertheless, it must be noted that our predictions are entirely based on *in-silico* functional annotation analysis and, therefore, further laboratory research is necessary to confirm the heavy metal resistance of this bacterium.

### 3.6. Genomic information unveils a *salt-out* osmoregulation strategy

Two main osmoregulatory mechanisms allow prokaryotes to dwell in high-salt concentration environments. The *salt-in* strategy is typical of extreme halophiles, mostly haloarchaea (Youssef et al., 2014) and some bacteria, such as those of the widely studied genus *Salinibacter* (Antón et al., 2002). To maintain the structure and activity of their proteins under high cytoplasmic ion concentrations these microorganisms possess a more acidic proteome (Oren, 2008). However, this kind of proteome has also been described in microorganisms with a *salt-out* mechanism (Elevi Bardavid and Oren, 2012; Oren, 2013), which is the most extended strategy across prokaryotes allowing adaptation to wider variations in the surrounding osmotic pressure (Oren, 2008).

**Figure 9:**
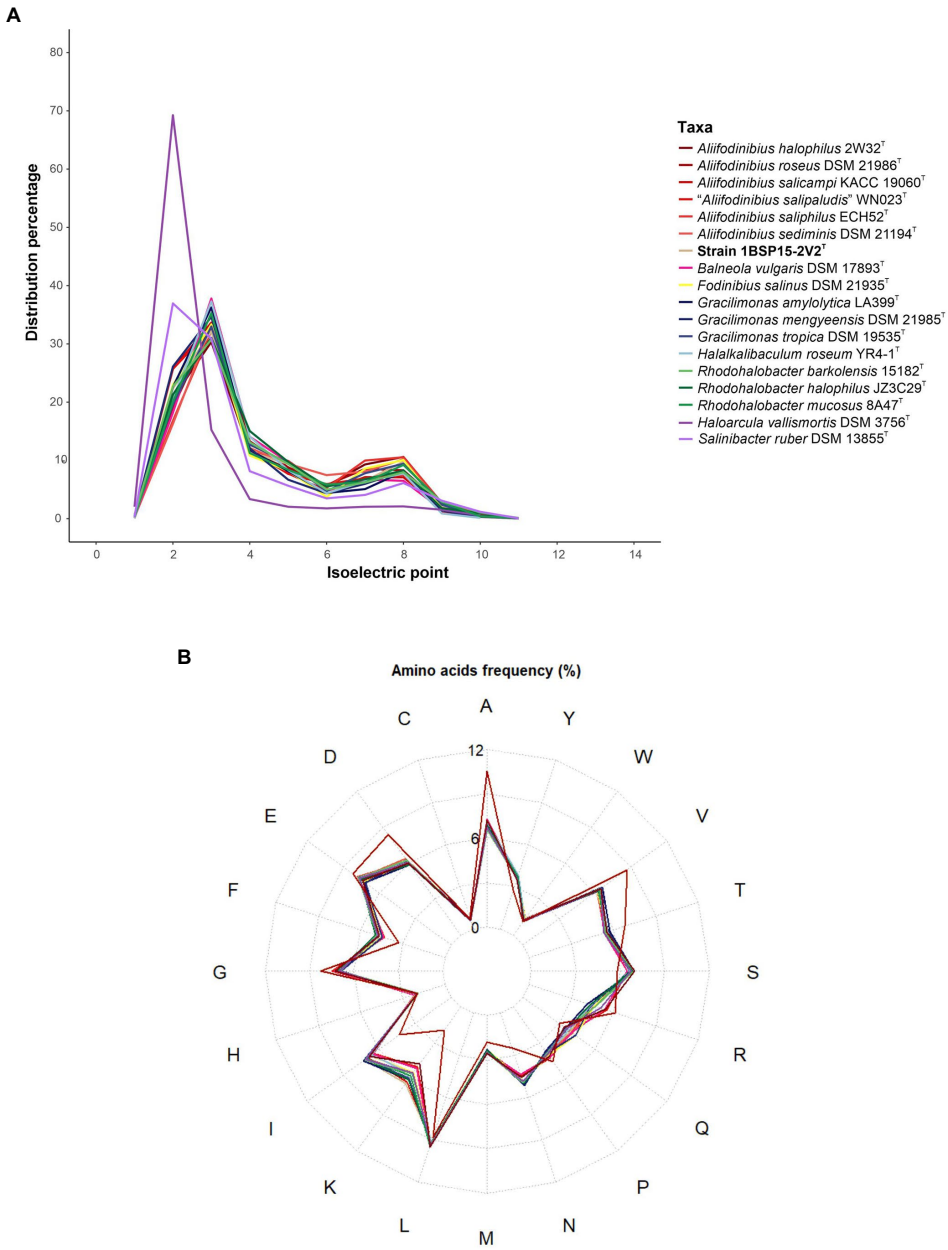
Isoelectric profile **(A)** and amino acid frequency **(B)** of the type strains of each species of the family *Balneolaceae*. The haloarchaeon *Haloarcula vallismortis* DSM 3756^T^ (GCF_900106715.1) and the bacterium *Salinibacter ruber* DSM 13855^T^ (GCF_000013045.1) have been included to compare our results with those of extreme halophiles with *salt-in* osmoregulatory mechanism. The genomes under study possessed a less acidic proteome, suggesting a *salt-out* strategy, though further *in vivo* analyses are necessary to confirm this hypothesis.

All genomes under study have a similar isoelectric profile (Figure 9A) and amino acid frequency distribution (Figure 9B), showing a less acidic proteome than those of the extreme haloarchaeon *Haloarcula vallismortis* and the bacterium *Salinibacter ruber*. Concerning the genome of strain 1BSP15-2V2^T^, it harbors Ktr potassium importer genes (K03498, K03499) that may contribute to a fast response against osmotic stress, as it has been previously observed in *Bacillus subtilis* (Holtmann et al., 2003; Hoffmann and Bremer, 2016) and *Synechocystis* sp. (Zulkifli et al., 2010). Another ion homeostasis-related protein annotated in the genome of strain 1BSP15-2V2^T^ (K03313) is the NhaA Na^+^/H^+^ antiporter, which plays a relevant role to avoid the toxicity of high cytoplasmatic Na^+^ concentrations (Krulwich et al., 2011; Patiño-Ruiz et al., 2022). However, potassium and sodium fluxes are impractical mechanisms to deal with sustained high osmolarity (Hoffmann and Bremer, 2017). For that reason, strain 1BSP15-2V2^T^ also presents compatible solute transporters of the Opu family (K05845, K05846, K05847), involved in the ATP-dependent uptake of a wide variety of compatible solutes into the cytoplasm, such as glycine betaine, choline, and proline betaine (Hoffmann and Bremer, 2017; Teichmann et al., 2018). Sudden decrease of osmolarity could also trigger stress in bacteria. Accordingly, strain 1BSP15-2V2^T^ encodes Msc mechanosensitive channels (K03282, K03442) which release ions and organic molecules if an osmotic down shock occurs (Booth and Blount, 2012; Booth, 2014). The different strategies to balance the osmotic pressure encoded in the genome of strain 1BSP15-2V2^T^ (Supplementary Table S5) indicate that this microorganism can thrive on a range of salt concentrations, which is consistent with the physiological traits tested under laboratory conditions (Supplementary Table S4). Consequently, strain 1BSP15-2V2^T^ can be defined as a moderately halophilic bacterium with a *salt-out* osmoregulation mechanism.

### 3.7. Members of the family *Balneolaceae* prefer saline soil versus aquatic hypersaline environments

**Figure 10:**
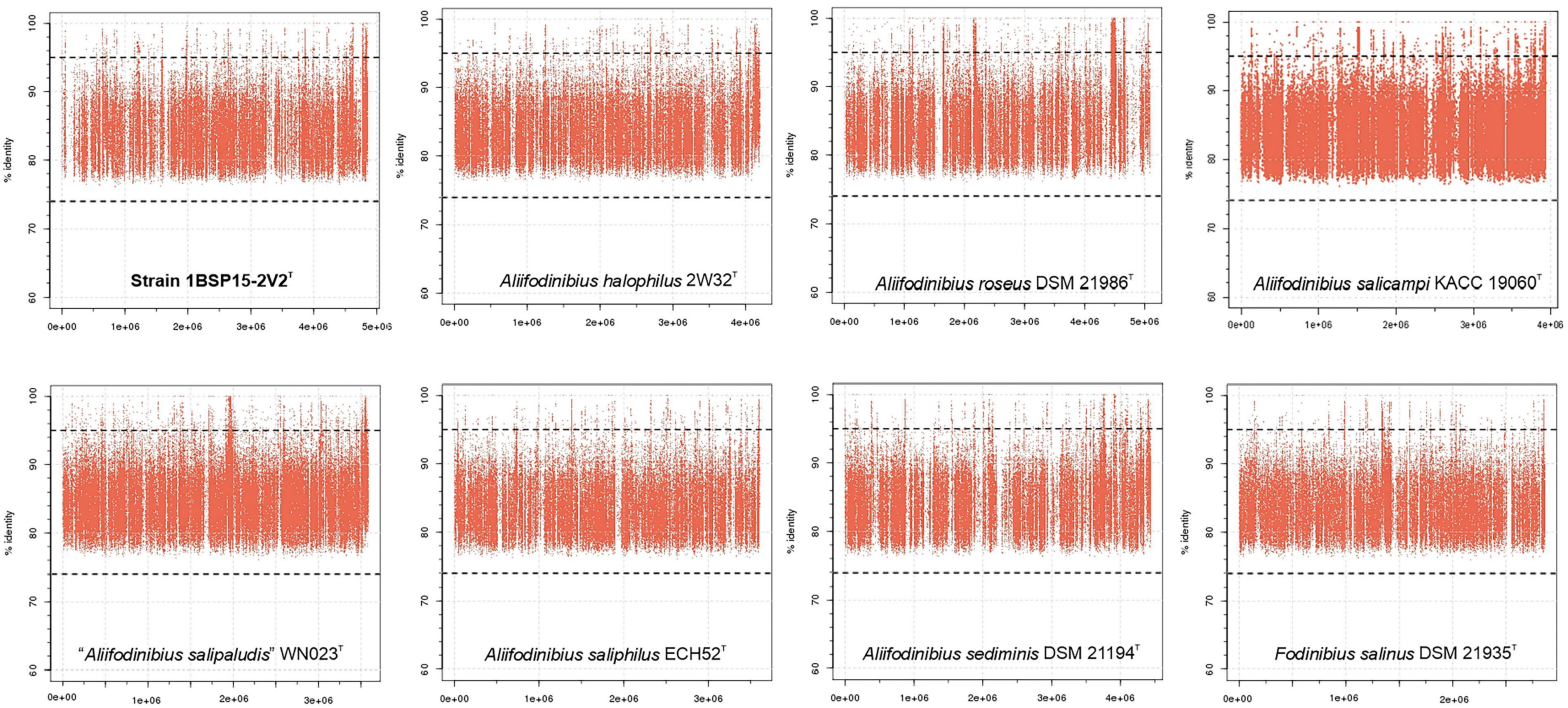
Genomic recruitment plots for the isolated strain 1BSP15-2V2^T^ and other type strains of species of the genera *Aliifodinibius* and *Fodinibius* against SMO1 hypersaline soil metagenomic reads. X-axis represents the genome length and Y-axis represents the percentage of identity of the reads matching the genome. Red dots above the 95% threshold line indicate read assignment at the species level. As it can be seen, most of the reads matches the genome at 80%–90% identity (above the proposed 73.98% for genus delineation), meaning they cannot be affiliated to any of the described *Aliifodinibius*/*Fodinibius* species, but they probably belong to a closed relative well represented in the SMO1 hypersaline soil.

**Figure 11:**
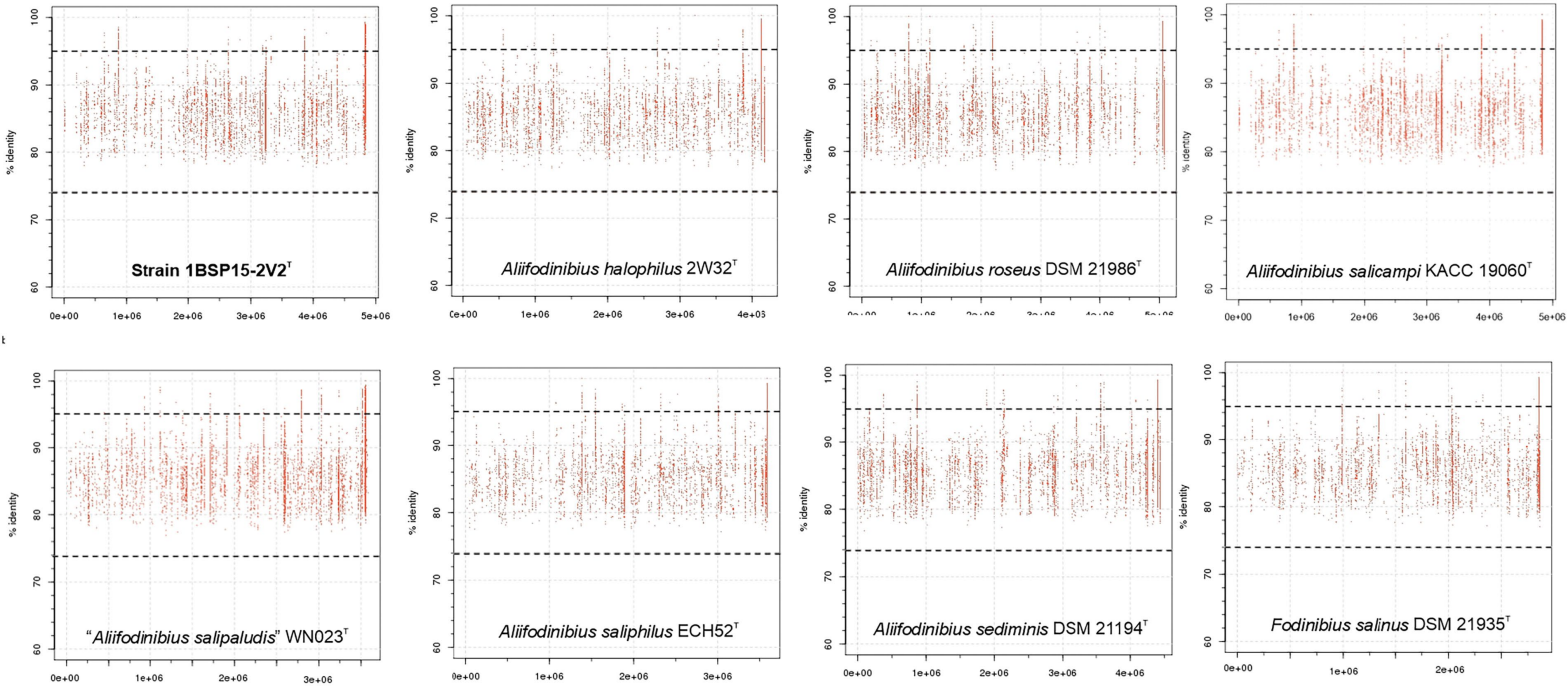
Genomic recruitment plots for the isolated strain 1BSP15-2V2^T^ and other type strains of species of the genera *Aliifodinibius* and *Fodinibius* against SS13 saltern metagenomic reads. X-axis represents the genome length and Y-axis represents the percentage of identity of the reads matching the genome. Red dots above the 95% threshold line indicate read assignment at the species level. A genus delineation line is also shown at 73.98% identity. The scarce read recruitment by all the species analyzed demonstrates their low abundance in this aquatic environment.

Genomic fragment recruitment analysis was performed against nine metagenomic datasets (Supplementary Table S2) to assess the environmental distribution of strain 1BSP15-2V2^T^ and the other species of the family *Balneolaceae*. SMO1 and SMO2 metagenomes come from the same hypersaline soils used in this study. The other seven metagenomic databases originated from hypersaline aquatic environments (salterns and lakes) were included in this study to determine the distribution of the new taxon in different saline habitats (soils and water). The selected datasets ranged from 13 to 37% (w/v) salt concentration. Of these, SS13 metagenome has the closest salinity (13% [w/v] salts) to the optimal salt concentration defined for strain 1BSP15-2V2^T^ (9% [w/v] salts). Our results show few reads (<0.01%) recruited at ≥95% identity from all species of the genera *Aliifodinibius*/*Fodinibius* for all metagenomic datasets. As nucleotide sequences are being compared, the 95% ANI threshold is considered for species delineation. Hence, the isolated species seems to be not highly abundant in the studied environments. However, soil and aquatic environments seem to follow different patterns, with a huge read recruitment at 80–90% identity for SMO1 and SMO2 (soils) datasets (Figure 10) not observed in water metagenomes (Figure 11). Barco et al. (2020) proposed an ANI cutoff value of 73.98% for genus demarcation, and thus, on this basis, it would suggest the existence of a taxon or group of taxa very closely related to the genera *Aliifodinibius* and *Fodinibius* (that could represent different species or even genera) abundant in those hypersaline soil environments. On the contrary, the scarce recruitment at 80–90% identity for the aquatic hypersaline databases might indicate that closed relatives of the genera *Aliifodinibius*/*Fodinibius* have a preference for hypersaline soils over their aquatic counterparts.

## 4. Conclusion

Phylogenetic and genomic evidence, fatty acid profiles, and phenotypic characteristics of the microorganisms under study show unequivocally that the species of the genera *Aliifodinibius* and *Fodinibius* should be placed into a single genus. We propose the reclassification of the members of the genus *Aliifodinibius* into the genus *Fodinibius*, since the genus name *Fodinibius* was validly published first and, thus, it has priority over the genus name *Aliifodinibius*, according to the International Code of Nomenclature of Prokaryotes (Parker et al., 2019). Additionally, strain 1BSP15-2V2^T^ represents a new species of the genus *Fodinibius*, for which the name *Fodinibius salsisoli* sp. nov. is proposed. The description of the new species, as well as the reclassification of the species of *Aliifodinibius* into the genus *Fodinibius* and, consequently, the emended description of the genus *Fodinibius* are enclosed below.

### Description of *Fodinibius salsisoli* sp. nov.

*Fodinibius salsisoli* (sal.si.so’li. L. masc. adj. *salsus* salty; L.; neut. n. *solum* soil; N.L. gen. n. *salsisoli* of salty soil).

Cells are Gram-stain-negative, non-endospore-forming, non-motile rods with a size of 0.05–0.10 × 0.20–0.30 μm. Colonies are irregular, opaque, and orange-red pigmented in SMM medium supplemented with 7.5% (w/v) salts after 5 days of incubation at 37°C. Grows between 3 and 20% (w/v) salt concentration (optimally at 9% [w/v]), pH 5.0–8.0 (optimally at pH 6.0), and 14–43°C (optimally at 37°C). Growth is not observed under anaerobic conditions. Catalase positive and oxidase negative. Reduces nitrate and nitrite. Positive for methyl red test but negative for Voges-Proskauer test, indicating that it uses the mixed acid pathway for glucose fermentation. Simmons’ citrate and phenylalanine deaminase are positive. Aesculin is hydrolyzed, but casein, DNA, gelatin, Tween 80, starch, and urea are not. H_2_S is produced, but not indole. Acids are produced from D-arabinose, D-fructose, D-glucose, maltose, sucrose, and D-xylose but not from D-galactose, glycerol, lactose, mannitol, and D-trehalose. Utilizes amygdaline, D-cellobiose, D-fructose, D-galactose, D-glucose, D-lactose, D-maltose, D-mannose, D-melezitose, ribose, D-raffinose, salicin, sucrose, D-trehalose, D-xylose, dulcitol, ethanol, glycerol, mannitol, D-sorbitol, xylitol, fumarate, hippurate, malate, propionate, and pyruvate as sole sources of carbon and energy, but not L-arabinose, starch, butanol, methanol, propranolol, acetate, benzoate, butyrate, citrate, formate, and valerate. Utilizes L-asparagine, aspartic acid, cysteine, L-glutamine, L-methionine, ornithine, L-phenylalanine, and L-serine as the sole sources of carbon, nitrogen, and energy, but not L-alanine, arginine, L-glutamate, glycine, L-isoleucine, lysine, L-threonine, tryptophan, and valine. The major fatty acids are iso-C_15:0_, C_16:1_ *ω*7c and/or C_16:1_ *ω*6c, and iso-C_17:1_ *ω*9c and/or 10-methyl C_16:0_. The genome of the type strain has an approximate size of 4.85 Mb and its G + C content is 44.5 mol%.

The type strain, 1BSP15-2V2^T^ (=CCM 9117^T^ = CECT 30246^T^), was isolated from hypersaline soils at the Odiel Saltmarshes Natural Area in Huelva (Southwest Spain). The accession number for the 16S rRNA gene sequence is MW811395 and that of the genome sequence GCF_026229185.1.

### Description of *Fodinibius halophilus* comb. nov.

Basonym: *Aliifodinibius halophilus* Xia et al. 2016.

The description is the same as for *Aliifodinibius halophilus* (Xia et al., 2016). The genome of the type strain has an approximate size of 4.19 Mb and its G + C content is 42.5 mol%. The accession number for the 16S rRNA gene sequence is KR559733 and that of the genome sequence GCF_011059105.1.

Type strain: 2W32^T^ (=CICC 23869^T^ = KCTC 42497^T^).

### Description of *Fodinibius roseus* comb. nov.

Basonym: *Aliifodinibius roseus* Wang et al. 2013.

The description is the same as for *Aliifodinibius roseus* (Wang et al., 2013). The genome of the type strain has an approximate size of 5.08 Mb and its G + C content is 48.3 mol%. The accession number for its 16S rRNA gene sequence is JQ923475 and that of the genome sequence GCF_900129315.1.

Type strain: YIM D15^T^ (=ACCC 10715^T^ = DSM 21986^T^ = KCTC 23442^T^).

### Description of *Fodinibius salicampi* comb. nov.

Basonym: *Aliifodinibius salicampi* Cho et al. 2017.

The description is the same as for *Aliifodinibius salicampi* (Cho et al., 2017, 2018). The genome of the type strain has an approximate size of 3.94 Mb and its G + C content is 42.8 mol%. The accession number for its 16S rRNA gene sequence is LC198077 and that of the genome sequence GCF_026228885.1.

Type strain: NBRC 112531^T^ (=KACC 19060^T^ = KHM44^T^).

### Description of *Fodinibius saliphilus* comb. nov.

Basonym: *Aliifodinibius saliphilus* Cho and Whang 2020.

The description is the same as for *Aliifodinibius saliphilus* (Cho and Whang, 2020). The genome of the type strain has an approximate size of 3.60 Mb and its G + C content is 40.8 mol%. The accession number for its 16S rRNA gene sequence is LC198072 and that of the genome sequence GCF_005869845.1.

Type strain: ECH52^T^ (=KACC 19126^T^ = NBRC 112664^T^).

### Description of *Fodinibius sediminis* comb. nov.

Basonym: *Aliifodinibius sediminis* Wang et al. 2013.

The description is the same as for *Aliifodinibius sediminis* (Wang et al., 2013). The genome of the type strain has an approximate size of 4.43 Mb and its G + C content is 48.1 mol%. The accession number for its 16S rRNA gene sequence is JQ923475 and that of the genome sequence GCF_900182555.1.

Type strain: YIM J21^T^ (=ACCC 10714^T^ = DSM 21194^T^).

### Emended description of the genus *Fodinibius* Wang et al. 2012

Cells are Gram-stain-negative, non-endospore-forming, non-motile rods, 0.05–1.5 μm in width and 0.2–5.5 μm in length (Wang et al., 2012). Colonies are salmon pink, pink, rose red, and reddish pigmented, circular, convex, and opaque with regular margins, and sometimes viscid. Some species are strictly aerobic bacteria, while others possess a facultatively anaerobic metabolism. Temperature range for growth between 14 and 45°C (optimum 37°C). Growth occurs at 3–25% (w/v) NaCl (optimum at 8–10%) and at pH values 5.0–10.0 (optimum at 6.0–8.0). All species produce catalase and most of them also oxidase. All species are unable to hydrolyze starch and DNA, and they cannot produce indole from tryptophane. Major fatty acids are iso-C_15:0_, iso-C_17:1_ *ω*9c, anteiso-C_15:0_, C_16:1_ *ω*7c and/or C_16:1_ *ω*6c, C_16:1_ *ω*7c and/or iso-C_15:0_ 2-OH, and iso-C_17:1_ *ω*9c and/or 10-methyl C_16:0_. The DNA G + C content ranges between 40.8–48.3 mol% (genome).

The type species is *Fodinibius salinus*. The genome of the type strain of this type species has an approximate size of 2.86 Mb and its G + C content is 42.5 mol%. The genus is member of the family *Balneolaceae*, order *Balneolales*, class *Balneolia*, phylum *Balneolota*.

## Data availability statement

The datasets presented in this study can be found in online repositories. The names of the repository/repositories and accession number(s) can be found at: https://www.ncbi.nlm.nih.gov/genbank/, JAGGJA000000000 https://www.ncbi.nlm.nih.gov/genbank/, JAJNDC000000000 https://www.ncbi.nlm.nih.gov/genbank/, MW811395.

## Author contributions

AV, CS-P, and RRH conceived the study. CG, CS-P, and AV acquired the environmental samples. CG performed the laboratory experiments and bioinformatic analysis, supported by CS-P and RRH, respectively. CG drafted the manuscript. CG, CS-P, RRH, and AV revised the manuscript. All authors contributed to the article and approved the submitted version.

## Funding

This study was supported by grants PID2020-118136GB-I00 funded by MCIN/AEI/10.13039/501100011033 and ERDF A way of making Europe, and from the Junta de Andalucía (P20_01066 and BIO-213), both with FEDER funds. CG was a recipient of a predoctoral fellowship (PRE2018-083242) from the Spanish Ministry of Science and Innovation. RRH was a recipient of a short-stay grant (PRX21/00598) from the Spanish Ministry of Universities.

## Conflict of interest

The authors declare that the research was conducted in the absence of any commercial or financial relationships that could be construed as a potential conflict of interest.

## Publisher’s note

All claims expressed in this article are solely those of the authors and do not necessarily represent those of their affiliated organizations, or those of the publisher, the editors and the reviewers. Any product that may be evaluated in this article, or claim that may be made by its manufacturer, is not guaranteed or endorsed by the publisher.

## Footnotes

^1^ https://www.ezbiocloud.net/

^2^ https://github.com/cristinagalisteo/gitana
