# Supplementary material for: Biotin pathway in novel Fodinibius salsisoli sp. nov., isolated from hypersaline soils and reclassification of the genus Aliifodinibius as Fodinibius
Source: Front Microbiol. 2023 Jan 26;13:1101464. doi: 10.3389/fmicb.2022.1101464 (PMC9909488; doi:10.3389/fmicb.2022.1101464)
Supplement: Supplementary file 1 [file Data_Sheet_1.pdf]

Table S1. Genomic features of members of the family *Balneolaceae* used in this study. Size, no. of contigs, N50, and G+C content were calculated by QUAST; completeness (%) and contamination (%) by CheckM; and no. of CDS, rRNA, tRNA, and CRISPRs by Prokka.

Strain	Accession number	Size (bp)	Contigs	N50	G+C (%)	Completeness (%)	Contamination (%)	CDS	rRNA	tRNA	CRISPRs
Strain 1BSP15-2V2^{T}	GCF_026229185.1	4,850,852	43	309,219	44.47	96.99	0.82	4,142	5	40	0
Aliifodinibius halophilus 2W32^{T}	GCF_011059105.1	4,188,534	80	149,403	42.52	98.09	0.82	3,680	2	39	0
Aliifodinibius roseus DSM 21986^{T}	GCF_900129315.1	5,082,244	64	195,789	48.33	96.99	0.82	4,267	2	42	0
Aliifodinibius salicampi KACC 19060^{T}	GCF_026228885.1	3,937,193	13	776,807	42.75	96.45	0.82	3,384	2	43	0
"*Aliifodinibius salipaludis*" WN023^{T}	GCF_002287075.1	3,583,276	39	310,580	42.24	97.54	2.46	3,143	2	39	0
Aliifodinibius saliphilus ECH52^{T}	GCF_005869845.1	3,606,883	11	1,898,413	40.84	97.81	1.37	3,139	2	40	2
Aliifodinibius sediminis DSM 21194^{T}	GCF_900182555.1	4,434,397	50	246,584	48.91	96.45	2.19	3,805	2	43	1
Balneola vulgaris DSM 17893^{T}	GCF_000375465.1	2,857,749	18	1,665,960	39.78	100.00	0.00	2,455	12	42	0
Balneola sp. EhC07	GCF_001650905.1	3,624,230	20	316,041	38.08	99.72	0.00	3,133	3	33	0
Fodinibius salinus DSM 21935^{T}	GCF_008124865.1	2,861,751	9	954,298	42.46	96.17	0.82	2,557	2	39	0
Gracilimonas amylolytica LA399^{T}	GCF_002911695.1	3,148,558	37	322,175	43.76	100.00	0.56	2,724	12	46	0
Gracilimonas mengyeensis DSM 21985^{T}	GCF_900182705.1	4,666,881	33	243,236	44.45	99.33	0.56	3,799	3	40	2
Gracilimonas tropica DSM 19535^{T}	GCF_000375425.1	3,831,242	48	280,641	42.95	99.89	0.00	3,347	6	42	0

Halalkalibaculum roseum YR4-1^T	GCF_011059145.1	3,829,494	21	1,000,808	44.02	97.54	0.82	3,259	2	40	0
Rhodohalobacter barkolensis 15182^T	GCF_002834295.1	3,597,295	7	1,807,017	42.48	97.54	1.37	3,025	4	40	0
Rhodohalobacter halophilus JZ3C29^T	GCF_001715195.1	3,122,722	92	84,288	44.43	98.09	0.82	2,750	4	38	1
Rhodohalobacter mucosus 8A47^T	GCF_003150675.1	3,908,029	17	398,890	47.72	98.09	1.37	3,263	3	41	0
Rhodohalobacter sp. 616A	GCF_021462415.1	4,693,446	10	1,181,382	42.25	97.54	0.82	3,947	3	43	0
Rhodohalobacter sp. SW132	GCF_017811715.1	5,083,342	45	392,673	44.76	97.81	1.37	4,144	3	40	0
"*Rhodohalobacter sulfatireducens*" WB101^T	GCF_022012475.1	5,104,032	138	123,641	41.85	97.54	1.37	4,286	5	38	0

Table S2. Metagenomic datasets used in this study along with their associated metadata.

Metagenomic dataset	Sample	Salt concentration	Size (Mb)	Average length (bp)	Accession number	Reference
SMO1	Hypersaline soil (Huelva, Spain)	24.0 mS/cm	867	629.0	SRR5753725	Vera-Gargallo et al. (2018)
SMO2	Hypersaline soil (Huelva, Spain)	54.4 mS/cm	507	543.0	SRR5753724	Vera-Gargallo et al. (2018)
IC21	Saltern (Isla Cristina, Spain)	21 % (w/v) NaCl	523	397.3	SRX352042	Fernández et al. (2014b)
SS13	Saltern (Alicante, Spain)	13 % (w/v) NaCl	501	302.2	SRX328504	Fernández et al. (2014a)
SS19	Saltern (Alicante, Spain)	19 % (w/v) NaCl	516	361.4	SRX090228	Ghai et al. (2011)
SS33	Saltern (Alicante, Spain)	33 % (w/v) NaCl	386	348.1	SRX347883	Fernández et al. (2014a)
SS37	Saltern (Alicante, Spain)	37 % (w/v) NaCl	331	418.2	SRX090229	Ghai et al. (2011)
Tyrrell_0.1	Hypersaline lake (Victoria, Australia)	29 % (w/v) NaCl	466	398.5	SRR5637210	Podell et al. (2014)
Tyrrell_0.8	Hypersaline lake (Victoria, Australia)	29% (w/v) NaCl	518	412.8	SRR5637211	Podell et al. (2014)

Table S3. Fatty acids composition of strain 1BSP15-2V2^T and other related species of the genera *Aliifodinibius* and *Fodinibius*.
1. Strain 1BSP15-2V2^T (this study); 2. *Aliifodinibius halophilus* 2W32^T; 3. *Aliifodinibius roseus* YIM D15^T; 4. *Aliifodinibius salicampi* KHM44^T; 5. "*Aliifodinibius salipaludis*" WN023^T; 6. *Aliifodinibius saliphilus* ECH52^T; 7. *Aliifodinibius sediminis* YIM J21^T; 8. *Fodinibius salinus* YIM D17^T. The major fatty acids (>10 %) are highlighted in bold. Fatty acids under 1 % present in all strains are omitted. -, Not detected.

Fatty acids	1	2[a]	3[b]	4[c]	5[d]	6[e]	7[f]	8[g]
$C_{16:0}$	2.2	2.2	2.6	2.8	2.8	-	2.1	3.7
$C_{18:0}$	-	-	0.4	-	-	-	0.7	1.4
Unknown $C_{13.565}$	-	2.5	-	-	-	-	-	-
iso-$C_{15:0}$	**36.4**	**25.5**	**19.2**	**29.5**	**33.3**	**19.2**	**16.0**	**23.6**
iso-$C_{15:1}$ F	0.7	-	1.1	-	-	-	1.2	2.4
iso-$C_{17:1}$ ω9c	-	**34.9**	-	**10.3**	**16.7**	**26.5**	-	-
iso-$C_{16:0}$	1.7	-	9	3.8	-	-	7.9	3.8
iso-$C_{17:0}$	0.9	3.1	1.6	2.0	-	-	2.3	0.3
iso-$C_{17:0}$ 3-OH	1.3	-	1.5	1.6	-	-	-	0.3
anteiso-$C_{15:0}$	8.2	1.4	**21.0**	7.4	5.8	-	**18.4**	8.1
anteiso-$C_{17:0}$	0.9	-	3.9	1.3	-	-	6.8	0.4
anteiso-$C_{17:0}$ ω9c	-	-	2.8	-	2.4	-	3.4	-
anteiso$C_{17:1}$ ω9c	-	-	-	1.0	-	-	-	1.4
$C_{15:1}$ ω6c	2.8	-	1.9	2.8	-	-	0.5	0.6
$C_{16:1}$ ω5c	5.1	3.8	2.3	2.3	2.5	-	2.9	1.5
$C_{17:1}$ ω6c	1.1	-	1.8	1.5	-	-	0.5	0.6
$C_{17:1}$ ω8c	1.4	-	1.7	2.2	-	-	0.3	0.5
$C_{18:1}$ ω9c	0.5	-	0.3	-	-	-	0.5	4.5
$C_{18:1}$ ω7c	-	-	0.3	-	-	-	2.9	-
10-methyl $C_{18:0}$	-	-	-	-	-	-	-	3.1
$C_{16:1}$ ω7c and/ or $C_{16:1}$ ω6c	**24.7**	-	**14.3**	-	-	-	**12.9**	**13.8**
$C_{16:1}$ ω7c and/or iso-$C_{15:0}$ 2-OH	-	**17.0**	-	**22.7**	**20.5**	**25.5**	-	-
iso-$C_{17:1}$ and/or $C_{17:1}$	-	1.4	-	-	1.6	-	-	-
iso-$C_{17:1}$ ω9c and/or 10-methyl $C_{16:0}$	**10.7**	-	**10.2**	-	-	-	**15.5**	**24.0**

[a] Xia et al. (2016). [b] Wang et al. (2013). [c] Cho et al. (2017). [d] Zhao et al. (2020). [e] Cho and Whang (2020). [f] Wang et al. (2013). [g] Wang et al. (2012).

Table S4. Differential physiological and biochemical characteristic among the species of the genera *Aliifodinibius* and *Fodinibius*.

1. Strain 1BSP15-2V2^T; 2. *Aliifodinibius halophilus* KCTC 42497^T; 3. *Aliifodinibius roseus* DSM 21986^T; 4. *Aliifodinibius salicampi* KACC 19060^T; 5. "*Aliifodinibius salipaludis"* KCTC 52855^T; 6. *Aliifodinibius saliphilus* KACC 19126^T; 7. *Aliifodinibius sediminis* DSM 21194^T; 8. *Fodinibius salinus* YIM D17^T. All strains tested in this study were positive for catalase, but negative for oxidase, hydrolysis of starch and DNA, production of indole, Simmons' citrate, Voges-Proskauer test, urease, phenylalanine deaminase, utilization of acetate, and valerate as sole carbon and energy sources, and utilization of L-alanine, arginine, L-glutamate, glycine, L-isoleucine, lysine, L-threonine, tryptophane, and valine as sole carbon, nitrogen, and energy sources. Strain *Fodinibius salinus* YIM D17^T was positive for catalase, hydrolysis of starch, and urease, but negative for indole production and utilization of acetate as sole carbon and energy source, according to its original description (Wang et al., 2012). ND, growth not detected; NA, not available; w, weakly positive result.

**Characteristic**	**1**	**2**	**3**	**4**	**5**	**6**	**7**	**8**[g]
NaCl range (%, w/v)	3-20	5-10[a]	2-18[b]	4-20[c]	3-25[d]	5-25[e]	5-25[f]	5-20
NaCl optimum (%, w/v)	9	9[a]	8[b]	8[c]	10[d]	10-25[e]	10[f]	10-15
pH range	5.0-8.0	5.0-8.0[a]	7.0-8.5[b]	6.5-8.0[c]	6.0-9.0[d]	6.5-11.0[e]	6.0-10.0[f]	6.0-9.0
pH optimum	6.0	6.0[a]	7.5-8.0[b]	7.0[c]	8.0[d]	7.5-8.0[e]	8.0[f]	7.5-8.0
Temperature range (°C)	14-43	14-43[a]	20-45[b]	20-42[c]	20-45[d]	15-45[e]	28-45[f]	25-45
Temperature optimum (°C)	37	37[a]	33-37[b]	28[c]	37[d]	33-37[e]	37[f]	37
Oxidase	-	-	-	-	-	-	-	+
Hydrolysis of gelatin	-	-	-	+	+	-	+	+
Hydrolysis of Tween 80	-	-	-	-	-	-	ND	+
Hydrolysis of aesculin	+	-	+	+	+	+	+	+
Hydrolysis of casein	-	ND	ND	-	ND	ND	ND	-
Methyl red test	+	-	-	-	+	+	+	NA
Nitrate reduction	+	+	-	-	+	-	-	+
Nitrite reduction	+	-	-	-	-	-	-	-
H_2S production	+	+	-	-	-	-	-	-
**Acid production from carbohydrates:**								
D-arabinose	+	-	+	+	+	+	+	NA
D-fructose	+	-	+	-	-	+	+	NA
D-galactose	-	-	+	-	-	-	+	NA
Glycerol	-	-	+	w	-	-	-	NA
D-glucose	+	-	+	+	+	+	+	NA
Lactose	-	-	+	+	-	-	-	NA
Maltose	+	-	-	+	+	+	+	NA
Mannitol	-	-	-	-	-	-	+	NA

Sucrose	+	-	+	+	-	-	+	NA
D-trehalose	-	-	+	+	-	-	+	NA
D-xylose	+	-	+	+	-	-	+	NA
**Utilization as sole carbon and energy source of:**								
Amygdalin	+	-	+	-	-	-	-	NA
L-arabinose	-	w	+	-	-	-	-	NA
D-cellobiose	+	-	+	-	-	-	-	NA
D-fructose	+	-	w	-	-	-	-	NA
D-galactose	+	-	w	-	-	-	-	-
D-glucose	+	-	+	-	-	-	-	-
D-lactose	+	-	+	-	-	-	-	-
D-maltose	+	-	+	-	-	-	w	-
D-mannose	+	-	+	-	-	-	w	-
D-melezitose	+	-	+	-	-	-	w	NA
Ribose	+	-	+	-	-	-	w	NA
D-raffinose	+	-	+	-	-	-	-	NA
Salicin	+	-	+	w	-	-	w	NA
Starch	-	-	+	-	-	-	-	NA
Sucrose	+	-	+	-	-	-	-	-
D-trehalose	+	-	+	w	w	-	w	NA
D-xylose	+	-	+	-	-	-	-	-
Butanol	-	-	+	-	-	-	-	NA
Dulcitol	+	w	+	-	-	-	-	NA
Ethanol	+	+	+	-	-	-	-	-
Glycerol	+	-	+	-	-	-	-	-
Mannitol	+	-	+	-	-	w	+	-
Methanol	-	-	+	-	-	-	-	-
Propranolol	-	-	+	-	-	-	-	NA
D-sorbitol	+	-	-	-	-	-	-	-
Xylitol	+	+	+	-	-	-	-	NA
Benzoate	-	-	+	-	-	-	-	NA
Butyrate	-	-	+	-	-	-	-	NA
Citrate	-	-	-	-	-	w	-	+
Formate	-	-	+	-	-	-	w	NA
Fumarate	+	-	+	-	-	-	-	NA
Hippurate	+	-	+	-	-	-	-	NA
Malate	+	-	+	-	-	-	-	NA
Pyruvate	+	+	+	-	-	-	-	NA
Propionate	+	-	w	-	-	-	-	NA

**Utilization as sole carbon, nitrogen, and energy source of:**								
L-asparagine	+	-	-	-	-	-	-	NA
Aspartic acid	+	-	-	-	-	-	-	-
Cysteine	+	-	-	-	-	-	-	NA
L-glutamine	+	+	-	+	-	+	-	NA
L-methionine	+	-	-	-	-	-	-	NA
Ornithine	+	-	+	+	-	-	-	NA
L-phenylalanine	+	-	-	-	-	-	-	NA
L-serine	+	+	+	w	-	-	-	NA

[a] Xia et al. (2016). [b] Wang et al. (2013). [c] Cho et al. (2017). [d] Zhao et al. (2020). [e] Cho and Whang (2020). [f] Wang et al. (2013). [g] Wang et al. (2012).

Table S5. Relevant KO numbers along with their definitions annotated for the genome of strain 1BSP15-2V2^T.

**Exclusive KO numbers**	
**KEGG ID**	**Definition**
K22616	olsC; ornithine lipid ester-linked acyl 2-hydroxylase [EC:1.14.11.58]
K02027	ABC.MS.S; multiple sugar transport system substrate-binding protein
K23247	oiaK; 3-oxoisoapionate kinase [EC:2.7.1.231]
K01559	oiaT; 3-oxoisoapionate-4-phosphate transcarboxylase/hydrolase [EC:3.7.1.28]
K18702	uctC; CoA:oxalate CoA-transferase [EC:2.8.3.19]
K07118	K07118; uncharacterized protein
K18298	mexE; membrane fusion protein, multidrug efflux system
K03576	metR; LysR family transcriptional regulator, regulator for metE and metH
K03923	mdaB; NADPH dehydrogenase (quinone) [EC:1.6.5.10]
K05841	E2.4.1.173; sterol 3beta-glucosyltransferase [EC:2.4.1.173]
K17837	bla2, blm, ccrA, blaB; metallo-beta-lactamase class B [EC:3.5.2.6]
K07126	K07126; uncharacterized protein
K19422	epsD; glycosyltransferase EpsD [EC:2.4.-.-]
K07005	K07005; uncharacterized protein
K01176	AMY, amyA, malS; alpha-amylase [EC:3.2.1.1]
K22684	MCA1; metacaspase-1 [EC:3.4.22.-]
K01193	INV, sacA; beta-fructofuranosidase [EC:3.2.1.26]
K08156	araJ; MFS transporter, DHA1 family, arabinose polymer utilization protein
K02638	petE; plastocyanin
K00244	frdA; succinate dehydrogenase flavoprotein subunit [EC:1.3.5.1]
K00245	frdB; succinate dehydrogenase iron-sulfur subunit [EC:1.3.5.1]
K00246	frdC; succinate dehydrogenase subunit C
K00247	frdD; succinate dehydrogenase subunit D
K00561	ermC, ermA; 23S rRNA (adenine-N6)-dimethyltransferase [EC:2.1.1.184]
**KO numbers related to pyruvate oxidation**	
**KEGG ID**	**Definition**
K00016	LDH, ldh; L-lactate dehydrogenase [EC:1.1.1.27]
K00024	mdh; malate dehydrogenase [EC:1.1.1.37]
K00027	ME2, sfcA, maeA; malate dehydrogenase (oxaloacetate-decarboxylating) [EC:1.1.1.38]
K00128	ALDH; aldehyde dehydrogenase (NAD+) [EC:1.2.1.3]
K00161	PDHA, pdhA; pyruvate dehydrogenase E1 component alpha subunit [EC:1.2.4.1]
K00162	PDHB, pdhB; pyruvate dehydrogenase E1 component beta subunit [EC:1.2.4.1]
K00244	frdA; succinate dehydrogenase flavoprotein subunit [EC:1.3.5.1]
K00245	frdB; succinate dehydrogenase iron-sulfur subunit [EC:1.3.5.1]
K00246	frdC; succinate dehydrogenase subunit C

K00247	frdD; succinate dehydrogenase subunit D
K00382	DLD, lpd, pdhD; dihydrolipoamide dehydrogenase [EC:1.8.1.4]
K00626	ACAT, atoB; acetyl-CoA C-acetyltransferase [EC:2.3.1.9]
K00627	DLAT, aceF, pdhC; pyruvate dehydrogenase E2 component (dihydrolipoamide acetyltransferase) [EC:2.3.1.12]
K00873	PK, pyk; pyruvate kinase [EC:2.7.1.40]
K01006	ppdK; pyruvate, orthophosphate dikinase [EC:2.7.9.1]
K01007	pps, ppsA; pyruvate, water dikinase [EC:2.7.9.2]
K01069	gloB, gloC, HAGH; hydroxyacylglutathione hydrolase [EC:3.1.2.6]
K01512	acyP; acylphosphatase [EC:3.6.1.7]
K01595	ppc; phosphoenolpyruvate carboxylase [EC:4.1.1.31]
K01610	E4.1.1.49, pckA; phosphoenolpyruvate carboxykinase (ATP) [EC:4.1.1.49]
K01649	leuA, IMS; 2-isopropylmalate synthase [EC:2.3.3.13]
K01679	E4.2.1.2B, fumC, FH; fumarate hydratase, class II [EC:4.2.1.2]
K01759	GLO1, gloA; lactoylglutathione lyase [EC:4.4.1.5]
K01895	ACSS1_2, acs; acetyl-CoA synthetase [EC:6.2.1.1]
K01961	accC; acetyl-CoA carboxylase, biotin carboxylase subunit [EC:6.4.1.2 6.3.4.14]
K01962	accA; acetyl-CoA carboxylase carboxyl transferase subunit alpha [EC:6.4.1.2 2.1.3.15]
K01963	accD; acetyl-CoA carboxylase carboxyl transferase subunit beta [EC:6.4.1.2 2.1.3.15]
K02160	accB, bccP; acetyl-CoA carboxylase biotin carboxyl carrier protein
K12972	ghrA; glyoxylate/hydroxypyruvate reductase [EC:1.1.1.79 1.1.1.81]
K13953	adhP; alcohol dehydrogenase, propanol-preferring [EC:1.1.1.1]
K13979	yahK; alcohol dehydrogenase (NADP+) [EC:1.1.1.2]
**KO numbers related to flagellar assembly**	
**KEGG ID**	**Definition**
K02386	flgA; flagellar basal body P-ring formation protein FlgA
K02387	flgB; flagellar basal-body rod protein FlgB
K02388	flgC; flagellar basal-body rod protein FlgC
K02389	flgD; flagellar basal-body rod modification protein FlgD
K02390	flgE; flagellar hook protein FlgE
K02391	flgF; flagellar basal-body rod protein FlgF
K02392	flgG; flagellar basal-body rod protein FlgG
K02393	flgH; flagellar L-ring protein FlgH
K02394	flgI; flagellar P-ring protein FlgI
K02396	flgK; flagellar hook-associated protein 1
K02397	flgL; flagellar hook-associated protein 3 FlgL
K02400	flhA; flagellar biosynthesis protein FlhA
K02401	flhB; flagellar biosynthesis protein FlhB
K02405	fliA, whiG; RNA polymerase sigma factor FliA
K02406	fliC, hag; flagellin
K02407	fliD; flagellar hook-associated protein 2
K02408	fliE; flagellar hook-basal body complex protein FliE
K02409	fliF; flagellar M-ring protein FliF

K02410	fliG; flagellar motor switch protein FliG
K02412	fliI; flagellum-specific ATP synthase [EC:7.4.2.8]
K02413	fliJ; flagellar protein FliJ
K02415	fliL; flagellar protein FliL
K02416	fliM; flagellar motor switch protein FliM
K02417	fliN; flagellar motor switch protein FliN
K02419	fliP; flagellar biosynthesis protein FliP
K02420	fliQ; flagellar biosynthesis protein FliQ
K02421	fliR; flagellar biosynthesis protein FliR
K02422	fliS; flagellar secretion chaperone FliS
K02556	motA; chemotaxis protein MotA
K02557	motB; chemotaxis protein MotB
K03086	rpoD; RNA polymerase primary sigma factor
K03092	rpoN; RNA polymerase sigma-54 factor
**KO numbers related to biotin metabolism**	
**KEGG ID**	**Definition**
K00059	fabG, OAR1; 3-oxoacyl-[acyl-carrier protein] reductase [EC:1.1.1.100]
K00208	fabI; enoyl-[acyl-carrier protein] reductase I [EC:1.3.1.9 1.3.1.10]
K00652	bioF; 8-amino-7-oxononanoate synthase [EC:2.3.1.47]
K00833	bioA; adenosylmethionine---8-amino-7-oxononanoate aminotransferase [EC:2.6.1.62]
K01012	bioB; biotin synthase [EC:2.8.1.6]
K01935	bioD; dethiobiotin synthetase [EC:6.3.3.3]
K02169	bioC; malonyl-CoA O-methyltransferase [EC:2.1.1.197]
K02372	fabZ; 3-hydroxyacyl-[acyl-carrier-protein] dehydratase [EC:4.2.1.59]
K03524	birA; BirA family transcriptional regulator, biotin operon repressor / biotin---[acetyl-CoA-carboxylase] ligase [EC:6.3.4.15]
K09458	fabF, OXSM, CEM1; 3-oxoacyl-[acyl-carrier-protein] synthase II [EC:2.3.1.179]
**KO numbers related to cobalamin transport**	
**KEGG ID**	**Definition**
K16092	btuB; vitamin B_{12} transporter
K25027	btuC; cobalamin transport system permease protein
K25028	btuD; cobalamin transport system ATP-binding protein [EC:7.6.2.8]
K25034	btuF; cobalamin transport system substrate-binding protein
**KO numbers related to folate biosynthesis**	
**KEGG ID**	**Definition**
K00287	DHFR, folA; dihydrofolate reductase [EC:1.5.1.3]
K00796	folP; dihydropteroate synthase [EC:2.5.1.15]
K00950	folK; 2-amino-4-hydroxy-6-hydroxymethyldihydropteridine diphosphokinase [EC:2.7.6.3]
K01077	E3.1.3.1, phoA, phoB; alkaline phosphatase [EC:3.1.3.1]
K01113	phoD; alkaline phosphatase D [EC:3.1.3.1]
K01633	folB; 7,8-dihydroneopterin aldolase/epimerase/oxygenase [EC:4.1.2.25 5.1.99.8 1.13.11.81]
K01665	pabB; para-aminobenzoate synthetase component I [EC:2.6.1.85]
K01724	PCBD, phhB; 4a-hydroxytetrahydrobiopterin dehydratase [EC:4.2.1.96]

K01737	queD, ptpS, PTS; 6-pyruvoyltetrahydropterin/6-carboxytetrahydropterin synthase [EC:4.2.3.12 4.1.2.50]
K03635	MOCS2B, moaE; molybdopterin synthase catalytic subunit [EC:2.8.1.12]
K03637	moaC, CNX3; cyclic pyranopterin monophosphate synthase [EC:4.6.1.17]
K03750	moeA; molybdopterin molybdotransferase [EC:2.10.1.1]
K03752	mobA; molybdenum cofactor guanylyltransferase [EC:2.7.7.77]
K08310	nudB, ntpA; dihydroneopterin triphosphate diphosphatase [EC:3.6.1.67]
K09007	folE2; GTP cyclohydrolase IB [EC:3.5.4.16]
K10026	queE; 7-carboxy-7-deazaguanine synthase [EC:4.3.99.3]
K11754	folC; dihydrofolate synthase / folylpolyglutamate synthase [EC:6.3.2.12 6.3.2.17]
K14652	ribBA; 3,4-dihydroxy 2-butanone 4-phosphate synthase / GTP cyclohydrolase II [EC:4.1.99.12 3.5.4.25]
**KO numbers related to pantothenate and CoA biosynthesis**	
**KEGG ID**	**Definition**
K00053	ilvC; ketol-acid reductoisomerase [EC:1.1.1.86]
K00128	ALDH; aldehyde dehydrogenase (NAD+) [EC:1.2.1.3]
K00606	panB; 3-methyl-2-oxobutanoate hydroxymethyltransferase [EC:2.1.2.11]
K00826	E2.6.1.42, ilvE; branched-chain amino acid aminotransferase [EC:2.6.1.42]
K00859	coaE; dephospho-CoA kinase [EC:2.7.1.24]
K00954	E2.7.7.3A, coaD, kdtB; pantetheine-phosphate adenylyltransferase [EC:2.7.7.3]
K01579	panD; aspartate 1-decarboxylase [EC:4.1.1.11]
K01652	E2.2.1.6L, ilvB, ilvG, ilvI; acetolactate synthase I/II/III large subunit [EC:2.2.1.6]
K01653	E2.2.1.6S, ilvH, ilvN; acetolactate synthase I/III small subunit [EC:2.2.1.6]
K01687	ilvD; dihydroxy-acid dehydratase [EC:4.2.1.9]
K01918	panC; pantoate--beta-alanine ligase [EC:6.3.2.1]
K03525	coaX; type III pantothenate kinase [EC:2.7.1.33]
K06133	LYS5, acpT; 4'-phosphopantetheinyl transferase [EC:2.7.8.-]
K13038	coaE62:F76hopantothenoylcysteine decarboxylase / phosphopantothenate---cysteine ligase [EC:4.1.1.36 6.3.2.5]
**KO numbers related to thiamine metabolism**	
**KEGG ID**	**Definition**
K00788	thiE; thiamine-phosphate pyrophosphorylase [EC:2.5.1.3]
K00939	adk, AK; adenylate kinase [EC:2.7.4.3]
K00941	thiD; hydroxymethylpyrimidine/phosphomethylpyrimidine kinase [EC:2.7.1.49 2.7.4.7]
K00946	thiL; thiamine-monophosphate kinase [EC:2.7.4.16]
K00949	thiN, TPK1, THI80; thiamine pyrophosphokinase [EC:2.7.6.2]
K01077	E3.1.3.1, phoA, phoB; alkaline phosphatase [EC:3.1.3.1]
K01662	dxs; 1-deoxy-D-xylulose-5-phosphate synthase [EC:2.2.1.7]
K03149	thiG; thiazole synthase [EC:2.8.1.10]

K03153	thiO; glycine oxidase [EC:1.4.3.19]
K03707	tenA; thiaminase (transcriptional activator TenA) [EC:3.5.99.2]
K04487	iscS, NFS1; cysteine desulfurase [EC:2.8.1.7]
K06949	rsgA, engC; ribosome biogenesis GTPase / thiamine phosphate phosphatase [EC:3.6.1.- 3.1.3.100]
**KO numbers related to nicotinate and nicotinamide metabolism**	
**KEGG ID**	**Definition**
K00135	gabD; succinate-semialdehyde dehydrogenase / glutarate-semialdehyde dehydrogenase [EC:1.2.1.16 1.2.1.79 1.2.1.20]
K00278	nadB; L-aspartate oxidase [EC:1.4.3.16]
K00322	sthA, udhA; NAD(P) transhydrogenase [EC:1.6.1.1]
K00324	pntA; H+-translocating NAD(P) transhydrogenase subunit alpha [EC:1.6.1.2 7.1.1.1]
K00325	pntB; H+-translocating NAD(P) transhydrogenase subunit beta [EC:1.6.1.2 7.1.1.1]
K00763	pncB, NAPRT1; nicotinate phosphoribosyltransferase [EC:6.3.4.21]
K00767	nadC, QPRT; nicotinate-nucleotide pyrophosphorylase (carboxylating) [EC:2.4.2.19]
K00858	ppnK, NADK; NAD+ kinase [EC:2.7.1.23]
K00969	nadD; nicotinate-nucleotide adenylyltransferase [EC:2.7.7.18]
K01081	E3.1.3.5; 5'-nucleotidase [EC:3.1.3.5]
K01950	E6.3.5.1, NADSYN1, QNS1, nadE; NAD+ synthase (glutamine-hydrolysing) [EC:6.3.5.1]
K03517	nadA; quinolinate synthase [EC:2.5.1.72]
K03742	pncC; nicotinamide-nucleotide amidase [EC:3.5.1.42]
K03783	punA, PNP; purine-nucleoside phosphorylase [EC:2.4.2.1]
K03787	surE; 5'/3'-nucleotidase [EC:3.1.3.5 3.1.3.6]
K08281	pncA; nicotinamidase/pyrazinamidase [EC:3.5.1.19 3.5.1.-]
K12410	npdA; NAD-dependent deacetylase [EC:2.3.1.286]
**KO numbers related to heavy metal resistance**	
K03325	ACR3, arsB; arsenite transporter
K03741	arsC; arsenate reductase (thioredoxin) [EC:1.20.4.4]
K03892	arsR; ArsR family transcriptional regulator, arsenate/arsenite/antimonite-responsive transcriptional repressor
K15725	czcC, cusC, cnrC; outer membrane protein, heavy metal efflux system
K15726	czcA, cusA, cnrA; heavy metal efflux system protein
K15727	czcB, cusB, cnrB; membrane fusion protein, heavy metal efflux system
K16264	czcD, zitB; cobalt-zinc-cadmium efflux system protein
K01534	zntA; Zn2+/Cd2+-exporting ATPase [EC:7.2.2.12 7.2.2.21]
**KO numbers related to osmoregulation**	
**KEGG ID**	**Definition**
K05845	opuC; osmoprotectant transport system substrate-binding protein
K05846	opuBD; osmoprotectant transport system permease protein
K05847	opuA; osmoprotectant transport system ATP-binding protein [EC:7.6.2.9]
K03498	trkH, trkG, ktrB, ktrD; trk/ktr system potassium uptake protein
K03499	trkA, ktrA, ktrC; trk/ktr system potassium uptake protein

K03313	nhaA; Na+:H+ antiporter, NhaA family
K03282	mscL; large conductance mechanosensitive channel
K03442	mscS; small conductance mechanosensitive channel

References

Cho, G. Y., Lee, J. C., and Whang, K. S. (2017). *Aliifodinibius salicampi* sp. nov., a moderately halophilic bacterium isolated from a grey saltern. *Int. J. Syst. Evol. Microbiol.* 67, 2598–2603. doi: 10.1099/ijsem.0.001981.

Cho, G. Y., and Whang, K. S. (2020). *Aliifodinibius saliphilus* sp. nov., a moderately halophilic bacterium isolated from sediment of a crystallizing pond of a saltern. *Int. J. Syst. Evol. Microbiol.* 70, 358–363. doi: 10.1099/ijsem.0.003765.

Fernández, A. B., Ghai, R., Martin-Cuadrado, A.-B., Sánchez-Porro, C., Rodriguez-Valera, F., and Ventosa, A. (2014a). Prokaryotic taxonomic and metabolic diversity of an intermediate salinity hypersaline habitat assessed by metagenomics. *FEMS Microbiol. Ecol.* 88, 623–635. doi: 10.1111/1574-6941.12329.

Fernández, A. B., Vera-Gargallo, B., Sánchez-Porro, C., Ghai, R., Papke, R. T., Rodriguez-Valera, F., et al. (2014b). Comparison of prokaryotic community structure from Mediterranean and Atlantic saltern concentrator ponds by a metagenomic approach. *Front. Microbiol.* 5, 196. doi: 10.3389/fmicb.2014.00196.

Ghai, R., Pašić, L., Fernández, A. B., Martin-Cuadrado, A.-B., Mizuno, C. M., McMahon, K. D., et al. (2011). New abundant microbial groups in aquatic hypersaline environments. *Sci. Rep.* 1, 135. doi: 10.1038/srep00135.

Podell, S., Emerson, J. B., Jones, C. M., Ugalde, J. A., Welch, S., Heidelberg, K. B., et al. (2014). Seasonal fluctuations in ionic concentrations drive microbial succession in a hypersaline lake community. *ISME J.* 8, 979–990. doi: 10.1038/ismej.2013.221.

Vera-Gargallo, B., Navarro-Sampedro, L., Carballo, M., and Ventosa, A. (2018). Metagenome sequencing of prokaryotic microbiota from two hypersaline soils of the Odiel salt marshes in Huelva, Southwestern Spain. *Genome Announc.* 6, e00140–e00118. doi: 10.1128/genomeA.00140-18.

Wang, Y. X., Liu, J. H., Xiao, W., Ma, X. L., Lai, Y. H., Li, Z. Y., et al. (2013). *Aliifodinibius roseus* gen. nov., sp. nov., and *Aliifodinibius sediminis* sp. nov., two moderately halophilic bacteria isolated from salt mine samples. *Int. J. Syst. Evol. Microbiol.* 63, 2907–2913. doi: 10.1099/ijs.0.043869-0.

Wang, Y. X., Liu, J. H., Xiao, W., Zhang, X. X., Li, Y. Q., Lai, Y. H., et al. (2012). *Fodinibius salinus* gen. nov., sp. nov., a moderately halophilic bacterium isolated from a salt mine. *Int. J. Syst. Evol. Microbiol.* 62, 390–396. doi: 10.1099/ijs.0.025502-0.

Xia, J., Ling, S. K., Wang, X. Q., Chen, G. J., and Du, Z. J. (2016). *Aliifodinibius halophilus* sp. nov., a moderately halophilic member of the genus *Aliifodinibius*, and proposal of *Balneolaceae* fam. nov. *Int. J. Syst. Evol. Microbiol.* 66, 2225–2233. doi: 10.1099/ijsem.0.001012.

Zhao, X., Miao, S., Sun, Y., Gong, Q., Zhao, J., Wang, J., et al. (2020). *Aliifodinibius salipaludis* sp. nov., isolated from saline-alkaline soil. *Curr. Microbiol.* 77, 1328–1333. doi: 10.1007/s00284-019-01863-w.
